# Strain and rate-dependent neuronal injury in a 3D *in vitro* compression model of traumatic brain injury

**DOI:** 10.1038/srep30550

**Published:** 2016-08-02

**Authors:** Eyal Bar-Kochba, Mark T. Scimone, Jonathan B. Estrada, Christian Franck

**Affiliations:** 1 Brown University, School of Engineering, Providence, RI, United States of America

## Abstract

In the United States over 1.7 million cases of traumatic brain injury are reported yearly, but predictive correlation of cellular injury to impact tissue strain is still lacking, particularly for neuronal injury resulting from compression. Given the prevalence of compressive deformations in most blunt head trauma, this information is critically important for the development of future mitigation and diagnosis strategies. Using a 3D *in vitro* neuronal compression model, we investigated the role of impact strain and strain rate on neuronal lifetime, viability, and pathomorphology. We find that strain magnitude and rate have profound, yet distinctively different effects on the injury pathology. While strain magnitude affects the time of neuronal death, strain rate influences the pathomorphology and extent of population injury. Cellular injury is not initiated through localized deformation of the cytoskeleton but rather driven by excess strain on the entire cell. Furthermore we find that, mechanoporation, one of the key pathological trigger mechanisms in stretch and shear neuronal injuries, was not observed under compression.

Traumatic brain injury (TBI) is a significant global health and economic challenge. In the United States alone, over 1.7 million people suffer from TBI every year^1^. At the cellular scale, diffuse neuronal injury, including diffuse axonal injury (DAI) and neurite swelling, has emerged as one of the hallmark pathological identifiers of TBI. Diffuse neuronal injury and DAI have been linked both to acute TBI symptoms^2^,^3^,^4^,^5^ and progressive neurodegenerative diseases, including Parkinson’s and Alzheimer’s disease^4^,^6^,^7^,^8^. Morphologically, diffuse neuronal injuries and cellular degeneration are identified by membrane swelling, or blebbing, due to vesicle and organelle accumulation within the injured neuronal cell^9^,^10^,^11^,^12^,^13^,^14^. Although the clinical importance of neuronal injury is widely recognized, a complete understanding of the exact strain and deformation mechanisms, modalities and thresholds producing the injury remains elusive. This challenge is compounded further by the lack of appropriate three-dimensional neuronal injury platforms capable of resolving the injury evolution with sufficient spatiotemporal detail. Previous *in vitro* investigations, derived largely from relatively stiff, two-dimensional substrata under the application of shear^15^,^16^,^17^,^18^,^19^ and stretch insults^20^,^21^,^22^,^23^,^24^,^25^, have shaped much of our current understanding of the injury mechanism, and its associate strain and strain-rate levels. While much of the literature has focused on investigations involving tensile and shear deformations, which can be straightforwardly recapitulated in simplified 2D assays, little spatiotemporal details of cellular injury strains have been reported of neurons undergoing compressive loading, which inherently requires 3D impact platforms. It is important to note that compressive deformations play a significant role in insults to the head as the majority of the initial impact-derived deformation wave is highly compressive^26^,^27^,^28^,^29^. Furthermore, result from head impact and finite element modeling simulations show that the brain is exposed to several compression waves during the course of a typical head impact^26^,^27^,^30^, yet detailed measurements correlating compressive strains to neuronal injury are largely lacking.

Furthermore, most *in-vitro* investigations of cellular TBI have provided discrete endpoint data, rather than detailing the temporal evolution of neuronal degeneration immediately post insult. Resolution of the injury time line as a function of applied strain and strain rate is important to determine the maximum window of possible therapeutic intervention to mitigate or prevent any irreversible cell damage. At a minimum, the timeline when neurite or axonal swelling is first observed should be documented as a function of impact strains and strain rate to this end.

To address this knowledge gap we developed a high-resolution, *in-situ* 3D neuronal compression device for administering controlled and repeatable uniaxial compression deformations to neurons embedded in 3D collagen gels. Since the device is installed directly on top of a laser scanning confocal microscope the entire continuous timeline of injury post-impact can be resolved. Using our previously-developed fast iterative digital volume correlation algorithm, we experimentally verified that the applied strain fields are indeed uniform throughout the entire imaging volume. By employing a custom-built 3D image segmentation procedure we resolve orientation-dependent neurite strains for strain magnitudes equal or greater than 0.012. Through spatiotemporal correlation between changes in neurite morphology and 3D strain mapping we find that excess in local shear strains correlate with the localization of neurite blebbing, but, interestingly, not with neuronal cell death. Instead, we find neuronal cell death to correlate most strongly with an excess in the total cell-averaged, or mean, axial strain nearly independent of applied strain rate. Strain rate on the other hand, had profound effects on neuronal pathomorphology and the extent of population injury. Lastly, we investigated whether compression-driven local axial and shear deformations would produce similar levels of mechanoporation generally observed in shear and tension mediated neuronal injury assays. Interestingly, under compression no significant amounts of mechanoporation were observed until cells became severely fragmented.

Given the prevalence of compressive deformations among TBI impacts, our findings provide new insight on the dependence of neuronal injury and pathomorpholgy on applied strain and strain rate in compression.

## Results

### 3D Compression model and characterization of the applied far-field strain

**Figure 1:**
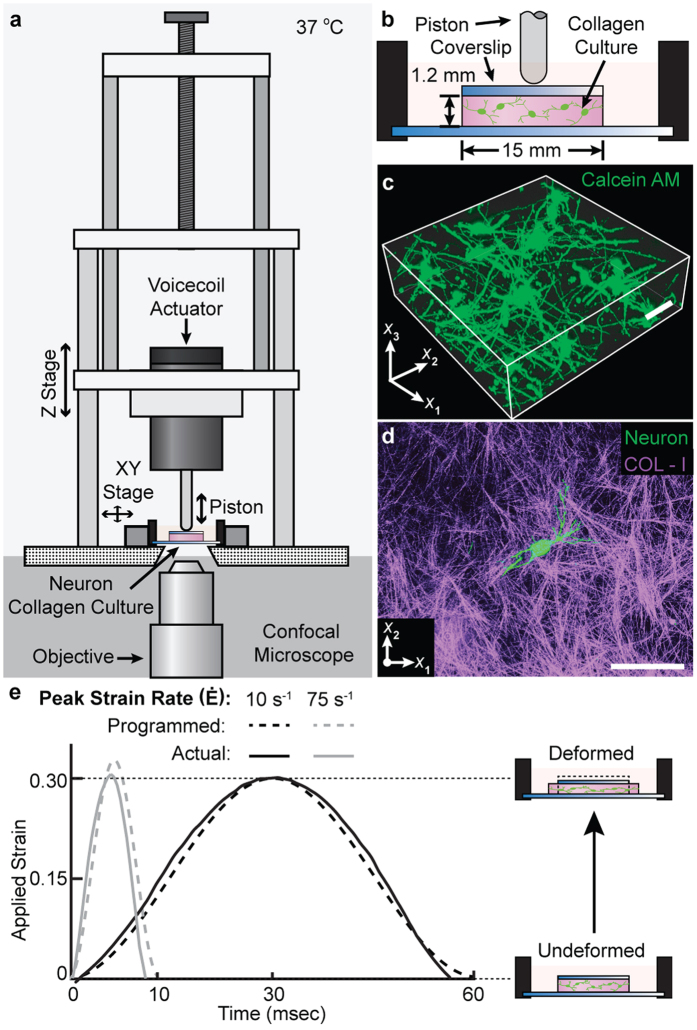
3D *in vitro* neuronal compression model. (**a**) Schematic representation of the cell compression device fitted onto a confocal microscope for spatiotemporal cell injury analysis. (**b**) Schematic representation of the 3D collagen culture system. Linear motion of the voice coil actuator piston drives the displacement of the cover slip, inducing far-field compressive deformation in the collagen culture. (**c**) Maximum intensity confocal micrographs of collagen culture at 7 days *in vitro*. Neurons are stained with calcein AM. Scale bar, 50 µm. (**d**) Maximum intensity projection of a neuron (green) encapsulated in a dense fibrillar collagen matrix (magenta) imaged with confocal reflectance microscopy. Scale bar, 50 µm. (**e**) Impact strain profiles generated by the cell compression device used in this study.

A new 3D cellular *in vitro* model of neuronal injury was developed to investigate the role of mechanoporation, shear, and axial deformations in neurons subjected to single compressive impacts. Primary cortical neurons embedded in type I collagen gels (2.2 mg/mL) were subjected to three different loading regimes (10^−4^ s^−1^, 10 s^−1^, and 75 s^−1^) at maximum compressive strains of 0.38 in quasistatic (10^−4^ s^−1^) and 0.30 in dynamic loading (Fig. 1a,b). The dynamically applied strain magnitudes of 0.30 and peak strain rates of 10 s^−1^ and 75 s^−1^ (Fig. 1e) were chosen to match the range of previous cellular TBI investigations^15^,^17^,^31^. Prior to loading, neuronal cultures exhibited morphologically healthy network formation (Fig. 1c) and viability (Supplementary Fig. S1) in the collagen matrix as determined with confocal microscopy (Fig. 1d), and compared to previous literature results^32^,^33^.

**Figure 2:**
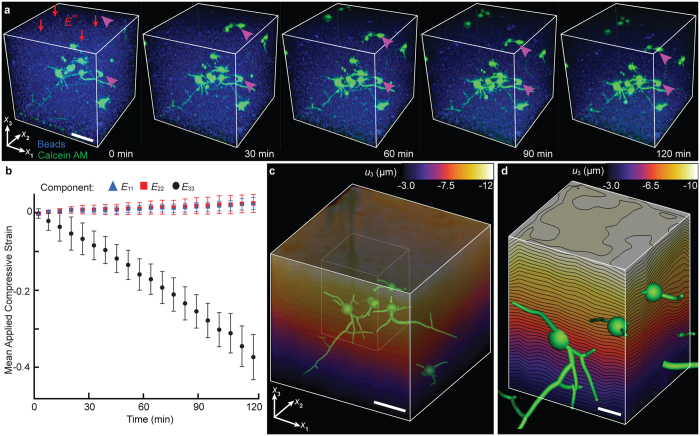
Characterization of the applied far-field compressive strain, *E*^∞^, under quasistatic (10^−4^) loading. (**a**) Maximum intensity confocal micrographs of a collagen gel embedded with neurons (green) and 0.5 µm fluorescent beads (blue) used to characterize the deformation field. Two neurons are tagged (magenta arrowheads) to show the incremental compression of the collagen. Scale bar, 50 µm. (**b**) Plot of the mean and standard deviation of the transverse (*E*_11_, blue triangle and *E*_22_, red square) and axial (*E*_33_, black circle) components of the mean Lagrangian strain tensor per loading increment as computed from the volumetric images shown in (**a**) using fast iterative digital volume correlation^34^. (**c**) Contour plot of the vertical displacement, *u*_3_, in the collagen matrix surrounding the neurons (green). Scal bar, 50 µm. (**d**) Zoomed-in view of the data subset (white box) shown in (**c**). Scale bar 20 µm.

In order to estimate the spatial distribution of matrix strains experienced by the embedded cells, the 3D volumetric strain fields inside the collagen gels were quantitatively resolved using our previously developed fast iterative digital volume correlation (FIDVC) technique^34^. Neuronal cultures embedded with 0.5 µm fluorescent microspheres were quasistatically compressed at applied far-field strain increments of 0.02 and subsequently imaged via confocal microscopy (Fig. 2a).

Quantitative assessment of the spatial distribution of the displacement field along the loading axis *x*_3_ shows that the collagen surrounding the neurons is uniformly compressed within the resolution of our FIDVC technique^35^, which, for the current study, can resolve any displacement motion greater than 0.63, 0.69, and 0.63 *μ*m in the *x*_1_, *x*_2_, and *x*_3_ directions, respectively (Fig. 2c,d; Supplemental Subsection: Spherical Inclusion Problem). Given the temporal limitation in typical laser scanning confocal microscopes, including our own, these 3D displacement measurements could only be carried out at quasistatic (10^−4^ s^−1^) conditions. While the stress response of both the collagen and neuronal networks may show significant viscoelastic effects^36^, the material strains, which are the controlled inputs of our device, are expected to be similar for the higher loading rates thus serving as estimators for the locally experienced cellular strain fields during impact. To determine whether fluid-derived shear stresses could be responsible for inducing neuronal injury at the higher loading rates, we visualized interstitial fluid flow through the collagen network using the tracer dye Rhodamine. We find that the average image intensity remained constant before and after impact showing that pressure driven flow (e.g., as described using Darcy’s law) was negligible for the collagen scaffolds used in our study, and that cell deformations were imparted primarily by the surrounding matrix rather than the pore fluid.

### Morphological injury assessment of neurons

**Figure 3:**
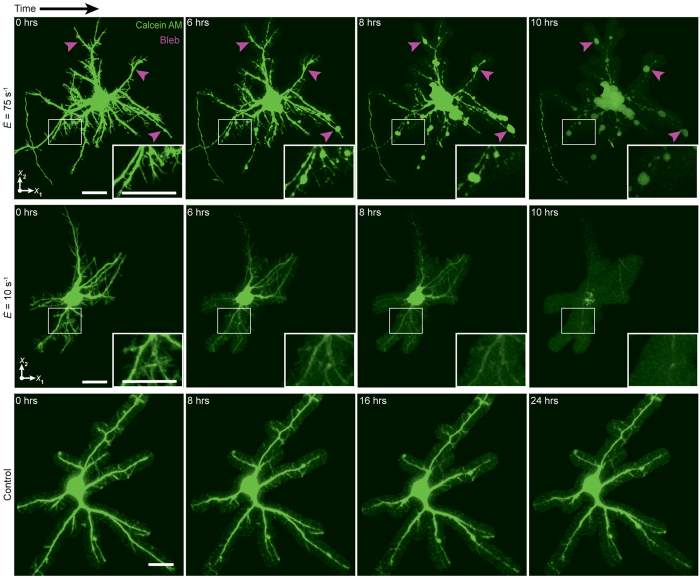
Morphological injury assessment after compressive impact. Maximum intensity projections of three representative neurons stained with calcein AM after mechanical loading at 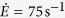 (top) and 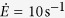 (middle), and a no-impact control. Arrows (magenta) indicate locations of bleb formation. Scale bars, 20 µm.

Common morphological TBI injury indicators including blebbing, retraction, and thinning of neurites were quantified at each strain rate via confocal time-lapse imaging. Time evolution 3D confocal micrographs of representative neurons for each strain rate revealed two distinct, loading-rate dependent neuropathomorphologies (Fig. 3). The first signs of structural degeneration, or changes within the pre-impact cytoskeleton, occured around six hours after the original impact. Once the structural changes became visible, cytoskeletal deteoriation proceeded quickly ending in cell death marked by either complete cellular fragmentation or somatic lysis around 9.7 hours. Somatic lysis is considered an endpoint criterion of cell death, in which failure of cellular structural components leads to termination of active cellular processes and necrosis^37^. Neurons impacted at 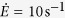 exhibited gradual retraction and thinning of their extensions, ultimately leading to somatic lysis and cell death (Fig. 3; middle row). Neurons impacted at 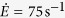 also exhibited this retraction and thinning morphology, but exhibited additional formation of visible focal blebs along their neurites. (Fig. 3; top row). Larger blebs for each cell formed at approximately the same time post-impact and grew in size until somatic lysis occurred. In contrast, smaller blebs gradually increased in number and size, producing local cell fragmentation. Quantifying the relative occurrence of each injury morphology for all impacted cells, we found an approximately 1.7-fold increase in occurrence of bleb formation with increasing strain rate: 37% of dead cells 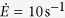 versus 64% formed blebs at 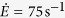. Compressed neurons at slow, quasi-static loading rates (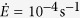) were indistinguishable from sham samples, which did not present any morphological changes (Fig. 3, bottom row) or changes in viability over the course of 24 hrs and beyond (Supplementary Fig. S1).

### Analysis of local and cell-averaged (mean) axial and shear strains

**Figure 4:**
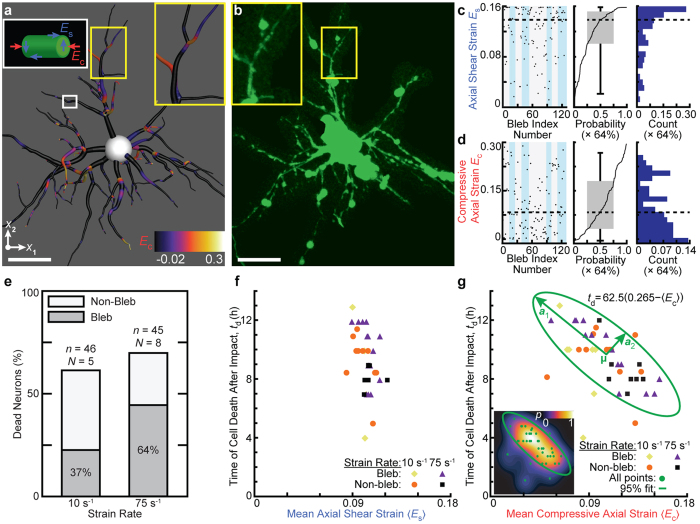
Analysis of the local and mean axial strains. (**a**) Computed local compressive axial strain *E*_*c*_ for a representative neuron. Scale bar, 20 µm. (**b**) Maximum intensity projection of the same neuron shown in (**a**) stained with calcein AM 8 hrs after impact at 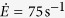 (bottom). Scale bar, 20 µm. (**c**) (left) Axial shear strain *E*_*s*_ at each bleb location for eight neurons at 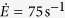. Alternate shading (light blue) pattern denotes data points for each cell at 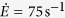. (center) Corresponding cumulative probability distribution function with box and whisker plot. Probability of finding a bleb given a certain value of *E*_*s*_ is multiplied by 64% (the percentage of bleb formation at 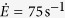). (right) Corresponding histogram normalized by total count. (**d**) The same computation in (**c**) for the compressive axial strain *E*_*c*_. (**e**) Stacked bar plots of the percentage of neuron death for 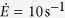 (*n* = 46, *N* = 5) and 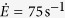 (*n* = 45, *N* = 8) for cells exhibiting non-bleb (white) and bleb (gray) formation. Dead neuron percentages were normalized by the 7 days *in vitro* control viability (see Supplementary Fig. S1). (**f**) Mean axial shear strain 〈*E*_*s*_〉 computed for all cells exhibiting bleb formation at 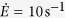 (yellow diamond, *n* = 7) and 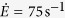 (purple triangle, *n* = 12), non-bleb formation at 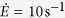 (orange circle, *n* = 14) and 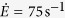 (black square, *n* = 8) as a function of time of cell death, *t*_*d*_. (**g**) Mean compressive axial strain 〈*E*_*c*_〉 computed for all cells exhibiting bleb formation at 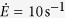 (yellow diamond, *n* = 7) and 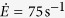 (purple triangle, *n* = 12), non-bleb formation at 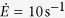 (orange circle, *n* = 14) and 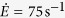 (black square, *n* = 8) as a function of time of cell death, *t*_*d*_. The ellipse (green) represents an isocontour at 95% confidence of the bivariate normal distribution fit with mean value *μ* and major and minor axes *a*_1_ and *a*_2_. (inset) Contour map of the bivariate probability density *p* of all data points (green circle).

To assess whether bleb formation is spatially correlated with a particular strain magnitude threshold, the local axial (*E*_*c*_) and shear (*E*_*s*_) strains were computed for neurons exhibiting clear bleb formation (Fig. 4a,b). The maximum value of *E*_*c*_ and *E*_*s*_ in the immediate proximity of each bleb was computed for eight neurons impacted at 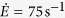 (Fig. 4c,d; Supplemental Methods). The inverse cumulative distribution function shows that bleb formation is strongly correlated with an average local shear threshold value of *E*_*s*_ = 0.14, while being uncorrelated with the axial strains. These data suggest that the presence of significant local shearing may generate cellular blebs within six to eight hours following the initial impact. An excess in local strains, however, may not be the primary cause of cell death, since only 32% of all dead cells presented neurite blebbing, and neither the local axial and shear strains correlated with other morphological features.

Thus instead, we computed the cell-averaged, or mean, axial and shear strains as a measure of the average strain distribution across the entire neuronal cytoskeleton. Given previous literature findings showing the existence and importance of a strain-dependent physical homeostasis in neurons^38^,^39^,^40^, we investigated whether a significant perturbation in this homeostasis would correlate with the temporal progression of neuronal degeneration.

Here, we plot the cell-averaged shear and axial strains for all recorded neurons versus the measured time of cell death, *t*_*d*_ (Fig. 4f,g; Supplemental Methods). Our results show that *t*_*d*_ strongly correlates with the mean axial strains but not the mean shear strains, highlighting the importance of injury modality during compression. Although, the result might seem obvious given that the far field strains are largely compressive, it is important to note that, because of the 3D orientation of each neurite within the gel, each cell encounters a significant amount of shear strain, which does not correlate with injury in our study.

The correlation between 〈*E*_*c*_〉 and *t*_*d*_ was quantified by computing the minimum covariance determinant (MCD) estimator, which fit the data to a bivariate normal distribution^41^,^42^. To confirm the fitting, the contour map of the bivariate probability density function shows a normal-like distribution within the ellipse (Fig. 4g (inset))^43^. The ellipse shown represents an isocontour at 95% confidence of the normal distribution and provides a relationship between time of cell death and the mean axial impact strain experienced. This relationship is described by the equation *t*_*d*_ = 62.5(0.265 − 〈*E*_*c*_〉), which signifies the major axis of the distribution and holds true within the 95% confidence interval. The ellipse is further characterized by the spatial location of the centroid *μ* (*t*_*d*_ = 9.67 hr, 〈*E*_*c*_〉 = 0.107) and major and minor axis lengths *a*_1_ (3.94 hr, 0.065) and *a*_2_ (1.65 hr, 0.014), respectively. The relative major and minor axis lengths define the eccentricity of the ellipse as 0.96.

### Cell permeability measurements

**Figure 5:**
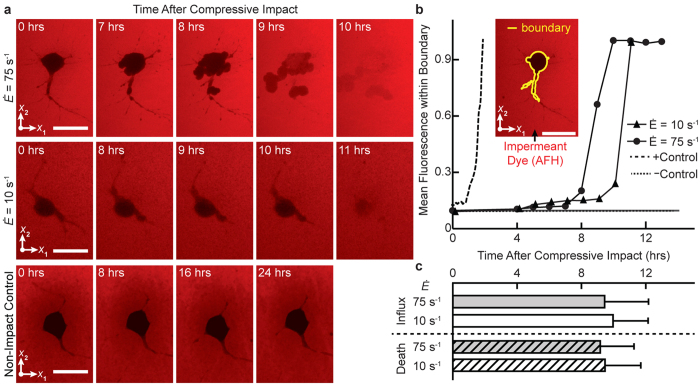
Cell permeability after compressive impact. (**a**) Minimum intensity projections of a representative neuron showing the spatiotemporal intensity distribution of cell-impermeant Alexa Fluor 568 hydrazide (AFH) after compression at strain rates 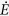 of 10 s^−1^ (top) and 75 s^−1^ (bottom). Scale bar, 20 µm. (**b**) Mean fluorescence intensity within the cell boundary (inset) for 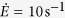 (solid triangle), 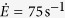 (solid circle), a positive control (long dash) with Triton X-100, and a negative control (short dash) without compressive loading. Scale bar, 20 µm. (**c**) AFH influx time (mean ± standard deviation), or peak rise in internal fluorescence, for 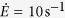 (solid white; *n* = 16 cells, *N* = 5 experiments) and 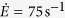 (solid gray; *n* = 23, *N* = 8), and cellular death time for 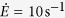 (hatched white; *n* = 19, *N* = 8) and 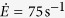 (hatched gray; *n* = 22, *N* = 8). No significant difference was found via t-test comparison.

Detection of mechanoporation was performed using the cell-impermeable fluorescent dye Alexa Fluor 568 hydrazide (AFH), which causes a rise in internal intensity upon passing through a compromised cell membrane (Fig. 5a). To quantitatively assess AFH influx after mechanical loading, the mean intensity within the cell boundary was computed for impact strain rates of 10 s^−1^ and 75 s^−1^. Mean intensity was compared to a positive control, treated with the surfactant Triton X-100, which generates significant membrane poration, and a negative control, without mechanical loading (Fig. 5b). The average influx time, defined as the time at which the internal fluorescence from AFH peaked (Fig. 5b), was compared to the average cellular death time for both strain rates (Fig. 5c). Time of cell death was assessed as the time point at which somatic lysis or a combination of leaking of live cell dye Calcein AM and severe morphological deformities occurred. Leakage of calcein AM fluorescence has been shown to correlate with other cell death markers^44^.

For loading rates of 10 s^−1^ and 75 s^−1^, AFH did not infiltrate the neurons until the cell membrane was already severely fragmented (Fig. 5a). This observation is contrary to several previous shear- and stretch-based *in vitro* injury models showing that membrane permeability steadily increases after impact loading^20^,^23^,^45^,^46^. Instead, we find that AFH inflow only occurred at the time of cell death with average influx times of 9.8 ± 2.2 h for 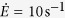 and 9.3 ± 2.7 h for 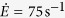, and average cell death times of 9.3 ± 2.2 h for 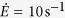 and 9.0 ± 2.1 h for 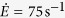 (Fig. 5c), which were statistically indistinguishable.

## Discussion

Through careful spatiotemporal analysis we determined our measured strain and strain rates to have profound, yet distinctly different effects on neuronal injury under compressive impact loading. In particular for the two evaluated impact loading rates (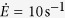, and 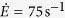), there was a marked difference in pathomorphologies experienced by the injured cells. The morphological differences are important for two reasons. First, it indicates a shift in neuropathomorphology with increasing strain rate, albeit an only small increase in the percentage of total cell death normalized by 7 day *in vitro* viability (Supplementary Fig. S1), i.e. 59% for 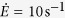 (*n* = 46 cells, *N* = 5 experiments) increasing to 67% for 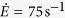 (*n* = 45 cells, *N* = 8 experiments) (Fig. 4e). Second, for impact durations on the order of 100 ms the observed neuronal injury morphologies less often include bleb formation, contrary to previously reported stretch and shear induced injuries^13^,^18^. This finding might be particularly important for histological screenings post-mortem, which may underpredict the injury extent if neurite blebbing is employed as the main pathomorphological indicator, which is not representative of strain rates at 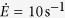. Although these slower strain rates may not reflect the time scale of the initial compression wave, it is conceivable that these slower loading rates exist within intracranial stress waves, which are likely to be attenuated by the various elastic impedance changes and viscosities present in brain matter^27^,^30^,^47^,^48^.

The observed bleb and cell death dependencies on strain rate (Figs 3 and 4e) may be due to the unique viscoelastic properties of the neuronal cytoskeleton^49^,^50^. In 2014 Ahmadzadeh *et al*. numerically estimated the rate-dependent disassociation of microtubule-associated tau proteins leading to neuronal injury at critical strain rates of 22–40 s^−1 ^31. At subcritical strain rates (i.e. <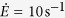), viscoelastic tau proteins allow for reversible sliding of microtubules^31^, preventing cellular damage, which is consistent with our quasistatic compression results (Figs 2 and 3) and previous literature results^51^,^52^. However, at supercritical rates, (e.g. 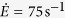), strain stiffening of tau proteins causes significant load transfer onto microtubules, resulting in large-scale microtubule damage^31^. This failure behavior at high strain rates may be responsible for disrupting neurite transport, causing the accumulation of vesicles and organelles, which manifests morphologically as bleb formation^13^.

Though striking, strain rate considerations only explain part of the injury narrative. While morphology during cell death has been determined, the timeline of cell death and the dependence on local versus mean axial strain requires attention. The histogram of local axial strain *E*_*c*_ shows a multimodal distribution, suggesting that bleb formation is not correlated with *E*_*c*_. In contrast, the histogram of *E*_*s*_ shows a skewed distribution with the highest peak at *E*_*s*_ ≈ 0.16, suggesting that local shear deformations may be the mechanical driving force behind bleb formation. However, it should be noted that bleb formation was only observed for a subset of all injured cells, and primarily occurred at the higher strain rate (Fig. 4e), suggesting that these local deformation measures cannot fully account for all observed cell deaths.

Recent studies have shown that long-range intra-axonal tension is required for the proper regulation and execution of neurotransmission^53^ and active vesicle transport along axons and neurites^54^. This tensional homeostasis is carefully regulated by neurons^50^ through the dynamic modulation of the cytoskeleton. Neurons via their internal contractility machinery tend to restore this physical homeostasis when perturbed on the time scales of minutes or longer^50^,^54^. Our results show that neurons subjected to large quasistatic compressive strains up to 0.38 show no sign of injury or cell death (Fig. 2). In contrast, neurons subjected to mechanical deformations at time scales much faster than their own contractility regulation times, as in the case for millisecond impacts (Fig. 3) may experience large-scale disruptions in tensional homeostasis resulting in neuronal injury and cell death. Furthermore, in our measurements we did not observe any noticeable rearrangement of neurons during the quasi-static loading and unloading cycle, suggesting that contractility rather than reorientation or shape changes are invoked to maintain physical homeostasis. This is consistent with previous literature results on other cell type showing that significant contractility and surface traction changes can occur without significant changes in cell shape^55^,^56^. However, it is important to note that cellular reorientation will eventually occur as a result of a significant alterations in cellular contractility given that the external load is not removed or that altered cell tractions are maintained over extended periods of time. To test whether a disruption in the physical homeostasis of the neuron is responsible for the observed cell death, we shifted our focus from resolving the local strain variations along neuronal extensions (Fig. 4c,d) to computing the mean (cell-averaged) shear and compressive axial strains experienced by each cell 〈*E*_*s*_〉 and 〈*E*_*c*_〉 (Fig. 4f,g), respectively. Interestingly, and independent of the investigated strain rates and bleb formation, all data points coalesced into a narrow band for both axial and shear strains. The general shape of each cluster indicates the functional correlation between *t*_*d*_ and the respective mean strain metric. The time of cell death was independent of the magnitude of the mean shear strain experienced by the cells, suggesting that cell death is not dependent upon 〈*E*_*s*_〉. In contrast, we found a strong correlation between the mean axial strain magnitude, 〈*E*_*c*_〉, and the time of cell death.

The relationship between mean axial strain and time of cell death defines the most likely timeframe of cell death after impact for a given mean axial strain magnitude. We found the relation between the mean axial strain, 〈*E*_*c*_〉, and time of cell death, *t*_*d*_ (Fig. 4g; Supplemental Methods) to be linear within an elliptical 95% isocontour of a bivariate normal distribution, or specifically, *t*_*d*_ = 62.5(0.265 − 〈*E*_*c*_〉). The high eccentricity of the ellipse (Fig. 4g) describes the linear behavior between the mean axial injury strain and time of cell death and suggests that cell death is primarily driven by the mean, not the local, compressive axial strain. The clustering of injury data points within the ellipse indicates that the relationship between the time of cell death and the level of experienced axial strain is independent of strain rate as long as the strain rate is sufficiently high (i.e. 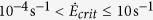). No cell death was observed for neurons undergoing compression at quasistatic (10^−4^ s^−1^) loading rates. The significance of this correlation is that it allows prediction of time of cell death for a given cell-average axial strain magnitude for a cell impacted above the critical strain rate. For example, neurons experiencing around 7% axial strain died on average 12 hours post impact, whereas neurons subjected to 12% strain died within only 7 hours. To our knowledge, this is the first 3D cellular death timeframe reported for neurons undergoing compression, and it presents a quantitative estimate on a potential therapeutic time window over which neuronal degeneration and subsequent cell death might be mitigated.

Finally, we investigated whether mechanoporation, which has been reported as an inciting event of neuronal injury in shear and tension assays, does also occur under compression. Interestingly and within the resolution of our confocal images (>200 nm), we did not observe mechanoporation in compression. The sharp, as opposed to gradual, increase in observed internal fluorescence (Fig. 5b) suggests that compression may not initiate the same enzymatic formation of non-resealable secondary pores (or mechanoporation), typically observed in stretch or shear deformation dominated neuronal injury models.

It is important to note that there are several limitations in our study, which should be addressed by future studies employing either the same or a similar experimental, high-resolution 3D approach. First, it is important to understand that many cells are sensitive to the local physical properties of their surrounding environment including the stiffness of the extracellular matrix, which may play an important role in the transduction of force and deformations onto the cells. Next, the role of integrins and their mechanotransducive capacity in neurons remains to be determined, which will have implications on the composition (e.g., laminin vs. collagen) and physical stiffness of the ECM. Given current advances in 3D cell matrix fabrication, these are all parameters that can be studied within the context of the presented experimental approach but are beyond the framework of the current study. Determination of the electrophysiology, spontaneous and stimulated neurotransmission pre- and post-impact provide exciting and important aspects for characterizing the full effect of the disease, but their current implementation particularly in 3D culture settings is still in its infancy. Approaches that are non-invasive and feature high spatiotemporal resolution are currently investigated and will provide additional insight into the temporal degeneration of neurons post-impact^57^. The strain rate effects on the presented neuronal pathormophology, while significant, are certainly not exhaustive, and future studies are encouraged to expand upon the rate-dependent results presented here. It should be noted that our current investigation focuses on examining the injury response of neurons subjected to a single compression cycle, rather than a more complex compression loading profile, which is consistent with currently available literature data. As new findings become available documenting actual brain tissue impact loading time histories, our experimental impact procedure can be straightforwardly modified to investigate the cellular injury response to more complex wave forms. Using the results of this study as a foundation, future studies should evaluate injury strain thresholds and associated correlations of the pathomorhology and cell death in neurons in mixed cell cultures including neurons, astroctyes and the various microglia^58^.

## Conclusion

This study presents previously undocumented spatiotemporal details on the strain and strain rate dependent pathomorphology of 3D neurons undergoing compressive impacts. Specifically, we show that neurite swelling, the morphological representation of cellular TBI, is highly loading rate-dependent in compression. That is, in regions of the brain undergoing compression, bleb formation associated with cellular TBI may represent just one pathological subset of neuronal injury, specifically, ones that have been compressed at high strain rates (e.g., 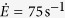). Injured tissue containing neurons not exhibiting bleb formation may therefore go undetected if neurite and axonal swelling are used as primary neuropathomorphology identifier, but may still represent an important source of cellular TBI.

Through careful spatiotemporal 3D analysis, we provide the first quantitative description of the local and mean, i.e., cell-averaged, injury strains and associated death timeframe for neurons under compression. Interestingly, we find that this injury timeframe was only a function of the applied strain magnitude, rather than the rate of strain. Conversely, strain rate dictated the extent of injured cells across the population, and as discussed earlier, significantly influenced the pathomorphology of neuronal injury, rendering both strain metrics important injury effectors. Lastly, we show that mechanoporation, a common event preceding neuronal degeneration in tension and shear TBI assays, is absent in compression for our applied strain magnitudes and strain rates.

Given the prevalence of compressive loading during head impacts, the findings of this study should provide beneficial information for the detection and quantification of neuronal injuries in *in vivo* investigations, clinical diagnosis, and simulation of head impacts^26^,^27^,^28^,^29^,^30^. Furthermore, albeit simplistic, this study may provide a foundation for quantitative experimental design to understand cellular thresholds and associated death timelines in 3D *in-vitro* culture models. Future studies should expand the current investigation to address how differences in extracellular matrix composition, co-culture conditions (e.g., inclusion of astrocytes), and mechanotransduction across ligand-integrin linkages affect the reported injury threshold and injury timeframes.

## Methods

### 3D Collagen Gel Preparation for Neuronal Compression

All procedures involving animals were approved by the Institutional Animal Care and Use Committee of Brown University and in accordance with the guidelines set forth. Cortical neurons were isolated from neonatal rats (Sprague-Dawley; p0-p1) and then encapsulated in 3D collagen (2.2 mg/mL) hydrogels. Cylindrical gels were cast using custom Delrin molds. Experiments were initiated after an initial 7 day *in vitro* (DIV) growth and synaptogenesis period, during which they were maintained in humidified cell incubators at 37 °C and 5% CO_2_. Culture medium was exchanged at 24 h and every 2 days thereafter.

### Confocal Microscopy and Live Cell Imaging

For all compression experiments, neuron viability and geometry were assessed using calcein AM (Life Technologies, Grand Island, NY), and acute alterations in plasma membrane permeability following compressive loading were assessed by evaluating uptake of Alexa Fluor 568 Hydrazide (AFH) (Life Technologies). Three-dimensional image volumes of neurons immediately after mechanical insult were acquired using a temperature-controlled Nikon A-1 confocal system mounted on a Ti-Eclipse inverted optical microscope controlled by NIS–Elements Nikon software (Nikon, Tokyo, Japan). Control experiments were conducted to confirm neuron population viability in the collagen hydrogels through 9 DIV (Supplementary Fig. S1) using calcein AM as a live cell stain and Ethidium homodimer–1 (EthD-1; Life Technologies) as a dead cell stain. Control experiments to induce cell membrane poration were performed by introducing 0.1% Triton X-100 after staining with calcein AM and AFH.

### 3D Cell Compression Device and Strain Field Characterization

The cell compression device consists of a custom frame-mounted linear voice coil actuator (VCA; LAS 13–18, BEI Kimco, San Marcos, CA) attached to the confocal microscope stage. Uniaxial compressive strain is generated by linear motion of the VCA piston in contact with a glass cover slip resting on top of the collagen gel. The piston has a spherical tip to ensure normal contact with the cover slip. Motion of the piston is produced via a servo controller (SilverSterling S2-IG; Quicksilver Controls, Inc., Covina, CA) and integrated hall-based displacement transducer. The VCA was programmed to deliver strain magnitudes of 0.30 and peak strain rates of 10 s^−1^ and 75 s^−1^. The uniaxial compressive strain field was validated under quasistatic loading (2% strain increments with 2 min loading time, to a maximum of 38%) on 3D collagen neuron cultures (n = 7) containing 6 v/v% fluorescent microspheres (0.5 µm diameter, Fisher Scientific) using fast-iterative digital volume correlation^34^.

### Quantitative injury Assessment

Strain in the local Frenet basis ***f***(*s*) = {***t***(*s*), ***n***(*s*), ***b***(*s*)} is dependent on orientation of the neurites and is calculated from the global far-field strain **E**^∞^ using the local rotation matrix, **Q**. The local strain **E**^(***f***)^ is calculated as


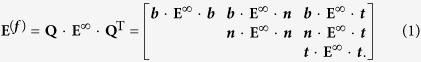


The local axial component of **E**^(***f***)^ is defined as *E*_*c*_ = ***t*** · **E**^∞^ · ***t*** and the local axial shear strain is defined as 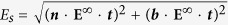. The mean axial strain 〈*E*_*c*_〉 and mean axial shear strain 〈*E*_*s*_〉 are then defined over the length of the neuron 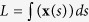 as


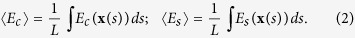


## Additional Information

**How to cite this article**: Bar-Kochba, E. *et al*. Strain and rate-dependent neuronal injury in a 3D *in vitro* compression model of traumatic brain injury. *Sci. Rep*. **6**, 30550; doi: 10.1038/srep30550 (2016).

## Supplementary Material

Supplementary Information

## References

[b1] Faul M., Xu L., Wald M. M., Coronado V. & Dellinger A. M. Traumatic brain injury in the United States: national estimates of prevalence and incidence. Inj. Prev. 16, A268 (2010).

[b2] Alexander M. P. Mild traumatic brain injury: Pathophysiology, natural history, and clinical management. Neurology 45, 1253–1260 (1995). 7617178 10.1212/wnl.45.7.1253

[b3] Inglese M. . Diffuse axonal injury in mild traumatic brain injury: a diffusion tensor imaging study. J. Neurosurg. 103, 298–303 (2005). 16175860 10.3171/jns.2005.103.2.0298

[b4] Johnson V. E., Stewart W. & Smith D. H. Axonal pathology in traumatic brain injury. Exp. Neurol. 246, 35–43 (2013). 22285252 10.1016/j.expneurol.2012.01.013 PMC3979341

[b5] Scheid R., Walther K., Guthke T., Preul C. & von Cramon D. Y. Cognitive sequelae of diffuse axonal injury. Arch. Neurol. 63, 418 (2006). 16533969 10.1001/archneur.63.3.418

[b6] Chen X.-H. . Long-term accumulation of amyloid-*β*, *β*-secretase, presenilin-1, and caspase-3 in damaged axons following brain trauma. Am. J. Physiol. 165, 357–371 (2004). 10.1016/s0002-9440(10)63303-2 PMC1618579 15277212

[b7] McKee A. C. . Chronic traumatic encephalopathy in athletes: progressive tauopathy after repetitive head injury. J. Neuropathol. Exp. Neurol. 68, 709–735 (2009). 19535999 10.1097/NEN.0b013e3181a9d503 PMC2945234

[b8] Johnson V. E., Stewart W. & Smith D. H. Traumatic brain injury and amyloid-*β* pathology: a link to Alzheimer’s disease? Nat. Rev. Neurosci. 11, 361–370 (2010). 20216546 10.1038/nrn2808 PMC3979339

[b9] Gaetz M. The neurophysiology of brain injury. Clin. Neurophysiol. 115, 4–18 (2004). 14706464 10.1016/s1388-2457(03)00258-x

[b10] Povlishock J. T. & Katz D. I. Update of neuropathology and neurological recovery after traumatic brain injury. J. Head Trauma Rehabil. 20, 76–94 (2005). 15668572 10.1097/00001199-200501000-00008

[b11] Okonkwo D. O. & Povlishock J. T. An intrathecal bolus of Cyclosporin A before injury preserves mitochondrial integrity and attenuates axonal disruption in traumatic brain injury. J. Cerebr. Blood. F. Met. 19, 443–451 (1999). 10.1097/00004647-199904000-00010 10197514

[b12] Farkas O. & Povlishock J. T. Cellular and subcellular change evoked by diffuse traumatic brain injury: a complex web of change extending far beyond focal damage. Prog. Brain Res. 161, 43–59 (2007). 17618969 10.1016/S0079-6123(06)61004-2

[b13] Kilinc D., Gallo G. & Barbee K. A. Mechanically-induced membrane poration causes axonal beading and localized cytoskeletal damage. Exp. Neurol. 212, 422–430 (2008). 18572167 10.1016/j.expneurol.2008.04.025

[b14] Hemphill M. A., Dauth S., Yu C. J., Dabiri B. E. & Parker K. K. Traumatic brain injury and the neuronal microenvironment: A potential role for neuropathological mechanotransduction. Neuron 85, 1177–1192 (2015). 25789754 10.1016/j.neuron.2015.02.041

[b15] Pfister B. J., Weihs T. P., Betenbaugh M. & Bao G. An *in vitro* uniaxial stretch model for axonal injury. Ann. Biomed. Eng. 31, 589–598 (2003). 12757202 10.1114/1.1566445

[b16] Lusardi T. A., Wolf J. A., Putt M. E., Smith D. H. & Meaney D. F. Effect of acute calcium influx after mechanical stretch injury *in vitro* on the viability of hippocampal neurons. J. Neurotraum. 21, 61–72 (2004). 10.1089/089771504772695959 14987466

[b17] Tang-Schomer M. D., Patel A. R., Baas P. W. & Smith D. H. Mechanical breaking of microtubules in axons during dynamic stretch injury underlies delayed elasticity, microtubule disassembly, and axon degeneration. FASEB J. 24, 1401–1410 (2010). 20019243 10.1096/fj.09-142844 PMC2879950

[b18] Hemphill M. A. . A possible role for integrin signaling in diffuse axonal injury. PLoS One 6, e22899 (2011). 21799943 10.1371/journal.pone.0022899 PMC3142195

[b19] Tang-Schomer M. D., Johnson V. E., Baas P. W., Stewart W. & Smith D. H. Partial interruption of axonal transport due to microtubule breakage accounts for the formation of periodic varicosities after traumatic axonal injury. Exp. Neurol. 233, 364–372 (2012). 22079153 10.1016/j.expneurol.2011.10.030 PMC3979336

[b20] Ellis E. F., McKinney J. S., Willoughby K. A., Liang S. & Povlishock J. T. A new model for rapid stretch-induced injury of cells in culture: Characterization of the model using astrocytes. J. Neurotraum. 12, 325–339 (1995). 10.1089/neu.1995.12.325 7473807

[b21] Zhang L., Rzigalinski B. A., Ellis E. F. & Satin L. S. Reduction of voltage-dependent Mg^2+^ blockade of NMDA current in mechanically injured neurons. Science 274, 1921–1923 (1996). 8943207 10.1126/science.274.5294.1921

[b22] Pike B. R. . Stretch injury causes calpain and caspase-3 activation and necrotic and apoptotic cell death in septohippocampal cell cultures. J. Neurotraum. 17, 283–298 (2000). 10.1089/neu.2000.17.283 10776913

[b23] Geddes D. M., Cargill R. S. & LaPlaca M. C. Mechanical stretch to neurons results in a strain rate and magnitude-dependent increase in plasma membrane permeability. J. Neurotraum. 20, 1039–1049 (2003). 10.1089/089771503770195885 14588120

[b24] Arundine M., Aarts M., Lau A. & Tymianski M. Vulnerability of central neurons to secondary insults after *in vitro* mechanical stretch. J. Neurosci. 24, 8106–8123 (2004). 15371512 10.1523/JNEUROSCI.1362-04.2004 PMC6729801

[b25] Geddes-Klein D. M., Schiffman K. B. & Meaney D. F. Mechanisms and consequences of neuronal stretch injury *in vitro* differ with the model of trauma. J. Neurotraum. 23, 193–204 (2006). 10.1089/neu.2006.23.193 16503803

[b26] Morse J. D., Franck J. A., Wilcox B. J., Crisco J. J. & Franck C. An experimental and numerical investigation of head dynamics due to stick impacts in girls’ lacrosse. Ann. Biomed. Eng. 42, 2501–2511 (2014). 25124650 10.1007/s10439-014-1091-8

[b27] Kraft R. H., Mckee P. J., Dagro A. M. & Grafton S. T. Combining the finite element method with structural connectome-based analysis for modeling neurotrauma: Connectome neurotrauma mechanics. PLoS Comput. Biol. 8, e1002619 (2012). 22915997 10.1371/journal.pcbi.1002619 PMC3420926

[b28] Mao H. . Finite element analysis of controlled cortical impact-induced cell loss. J Neurotraum. 27, 877–888 (2010). 10.1089/neu.2008.0616 PMC2943943 20199194

[b29] Young L. . When physics meets biology: low and high-velocity penetration, blunt impact, and blast injuries to the brain. Front. Neurol. Neurosci. 6 (2015). 10.3389/fneur.2015.00089 PMC4423508 25999910

[b30] Moore D. F. . Computational biology – modeling of primary blast effects on the central nervous system. Neuroimage 47, T10–T20 (2009). 19248833 10.1016/j.neuroimage.2009.02.019

[b31] Ahmadzadeh H., Smith D. H. & Shenoy V. B. Viscoelasticity of tau proteins leads to strain rate-dependent breaking of microtubules during axonal stretch injury: Predictions from a mathematical model. Biophys. J. 106, 1123–1133 (2014). 24606936 10.1016/j.bpj.2014.01.024 PMC4026781

[b32] Berthiaume F. & Morgan J. R. Methods in Bioengineering: 3D Tissue Engineering (Artech House, 2014).

[b33] Cullen D. K., Lessing M. C. & LaPlaca M. C. Collagen-dependent neurite outgrowth and response to dynamic deformation in three-dimensional neuronal cultures. Ann. Biomed. Eng. 35, 835–846 (2007). 17385044 10.1007/s10439-007-9292-z

[b34] Bar-Kochba E., Toyjanova J., Andrews E., Kim K.-S. & Franck C. A fast iterative digital volume correlation algorithm for large deformations. Exp. Mech. 55, 261–274 (2015).

[b35] Estrada J. B. & Franck C. Intuitive interface for the quantitative evaluation of speckle patterns for use in digital image and volume correlation techniques. J. Appl. Mech. 82, 095001 (2015).

[b36] Fratzl P. Collagen: structure and mechanics, Ch. 6 (Springer Science & Business Media, 2008).

[b37] Elmore S. Apoptosis: a review of programmed cell death. Toxicol. Pathol. 35, 495–516 (2007). 17562483 10.1080/01926230701320337 PMC2117903

[b38] Garland P. . Soluble axoplasm enriched from injured CNS axons reveals the early modulation of the actin cytoskeleton. PloS one 7, e47552 (2012). 23115653 10.1371/journal.pone.0047552 PMC3480358

[b39] Di Pietro V. . Transcriptomics of traumatic brain injury: gene expression and molecular pathways of different grades of insult in a rat organotypic hippocampal culture model. J Neurotraum. 27, 349–359 (2010). 10.1089/neu.2009.1095 19903084

[b40] Dubreuil C. I., Marklund N., Deschamps K., McIntosh T. K. & McKerracher L. Activation of rho after traumatic brain injury and seizure in rats. Exp. Neurol. 198, 361–369 (2006). 16448651 10.1016/j.expneurol.2005.12.002

[b41] Rousseeuw P. J. Least median of squares regression. J. Am. Stat. Assoc. 79, 871–880 (1984).

[b42] Rousseeuw P. J. & Driessen K. V. A fast algorithm for the minimum covariance determinant estimator. Technometrics 41, 212–223 (1999).

[b43] Botev Z., Grotowski J., Kroese D. . Kernel density estimation via diffusion. Ann. Stat. 38, 2916–2957 (2010).

[b44] Bratosin D., Mitrofan L., Palii C., Estaquier J. & Montreuil J. Novel fluorescence assay using Calcein AM for the determination of human erythrocyte viability and aging. Cytom. Part A 66, 78–84 (2005). 10.1002/cyto.a.20152 15915509

[b45] LaPlaca M. C., Cullen D., McLoughlin J. J. & Cargill R. S. High rate shear strain of three-dimensional neural cell cultures: a new *in vitro* traumatic brain injury model. J. Biomech. 38, 1093–1105 (2005). 15797591 10.1016/j.jbiomech.2004.05.032

[b46] Cullen D. K. & LaPlaca M. C. Neuronal response to high rate shear deformation depends on heterogeneity of the local strain field. J. Neurotraum. 23, 1304–1319 (2006). 10.1089/neu.2006.23.1304 16958583

[b47] Nyein M. K. . In silico investigation of intracranial blast mitigation with relevance to military traumatic brain injury. Proc. Natl. Acad. Sci. USA 107, 20703–20708 (2010). 21098257 10.1073/pnas.1014786107 PMC2996433

[b48] Pervin F. & Chen W. W. Dynamic mechanical response of bovine gray matter and white matter brain tissues under compression. J. Biomech. 42, 731–735 (2009). 19269640 10.1016/j.jbiomech.2009.01.023

[b49] Saif T., Rajagopalan J. & Tofangchi A. The role of mechanical tension in neurons. MRS Proceedings 1274 (2010).

[b50] Rajagopalan J., Tofangchi A. & Saif M. T. A. Drosophila neurons actively regulate axonal tension *in vivo*. Biophys. J. 99, 3208–3215 (2010). 21081068 10.1016/j.bpj.2010.09.029 PMC2980728

[b51] LaPlaca M. C. & Thibault L. E. An *in vitro* traumatic injury model to examine the response of neurons to a hydrodynamically-induced deformation. Ann. Biomed. Eng. 25, 665–677 (1997). 9236979 10.1007/BF02684844

[b52] Cullen D. K., Vernekar V. N. & LaPlaca M. C. Trauma-induced plasmalemma disruptions in three-dimensional neural cultures are dependent on strain modality and rate. J. Neurotraum. 28, 2219–2233 (2011). 10.1089/neu.2011.1841 PMC3218387 22023556

[b53] Siechen S., Yang S., Chiba A. & Saif T. Mechanical tension contributes to clustering of neurotransmitter vesicles at presynaptic terminals. Proc. Natl. Acad. Sci. USA 106, 12611–12616 (2009). 19620718 10.1073/pnas.0901867106 PMC2713391

[b54] Ahmed W. W. & Saif T. A. Active transport of vesicles in neurons is modulated by mechanical tension. Sci. Rep. 4 (2014). 10.1038/srep04481 PMC3967286 24670781

[b55] Toyjanova J., Flores-Cortez E., Reichner J. S. & Franck C. Matrix confinement plays a pivotal role in regulating neutrophil-generated tractions, speed, and integrin utilization. J. Biol. Chem. 290, 3752–3763 (2014). 25525264 10.1074/jbc.M114.619643 PMC4319039

[b56] Henry S. J., Crocker J. C. & Hammer D. A. Ligand density elicits a phenotypic switch in human neutrophils. Integr. Biol. 6, 348–356 (2014). 10.1039/c3ib40225h PMC5850933 24480897

[b57] Prevedel R. . Simultaneous whole-animal 3d imaging of neuronal activity using light-field microscopy. Nat. Methods 11, 727–730 (2014). 24836920 10.1038/nmeth.2964 PMC4100252

[b58] Dingle Y.-T. L. . Three-dimensional neural spheroid culture: An *in vitro* model for cortical studies. Tissue Eng. Pt. C: Meth. 21, 1274–1283 (2015). 10.1089/ten.tec.2015.0135 PMC4663656 26414693

