# Supplementary material for: Strain and rate-dependent neuronal injury in a 3D in vitro compression model of traumatic brain injury
Source: Sci Rep. 2016 Aug 2;6:30550. doi: 10.1038/srep30550 (PMC4969749; doi:10.1038/srep30550)
Supplement: Supplementary Information [file srep30550-s1.pdf]

Supplementary Information – Strain and rate-dependent neuronal injury in a 3D *in vitro* compression model of traumatic brain injury

Eyal Bar-Kochba[1], Mark T Scimone[1], Jonathan B Estrada[1], and Christian Franck[1,*]

[1]Brown University, School of Engineering, Providence, RI, United States of America
[*]franck@brown.edu

ABSTRACT

Materials & Methods

Isolation of Primary Cortical Neurons

All procedures involving animals followed the guidelines set forth and were approved by the Institutional Animal Care and Use Committee of Brown University. All reagents were purchased from Life Technologies (Grand Island, NY) unless otherwise noted. Primary rat cortical neurons were isolated from postnatal day 0-1 Sprague-Dawley rats (Charles River, Wilmington, MA). The pups were rapidly decapitated, and the cerebral hemispheres were placed in a cold solution of Hibernate A (HA; Brain Bits LLC, Springfield, IL) + 2% B-27 (50×) + 0.25% GlutaMAX (100×). Cerebral cortices were isolated and subsequently minced and digested in pre-warmed Hibernate A minus $CaCl_2$ (Brain Bits, LLC.) with Papain (2 mg/mL; Worthington Biochemical Corporation, Lakewood, NJ) for 30 minutes at 37°C with gentle agitation every 5 minutes. Half of the papain solution was replaced with pre-warmed HA/B-27 and centrifuged at 150g for 5 minutes. Dissociated tissue was resuspended in culture media (Neurobasal medium + 2% B-27 + 1% mixture of Penicillin Streptomycin, L-Glutamine, and Fetal Bovine Serum), and gently passed through a sterile cell strainer (40 µm mesh size; Thermo Fisher Scientific Inc., Waltham, MA) to filter large debris. Following another centrifuge step of 150g for 5 minutes, the cells were gently resuspended by mechanical trituration in culture media, and counted using a hemocytometer (Hausser Scientific, Horsham, PA). Cell solution was diluted with culture media to 10^7 cells/mL prior to preparation of 3D collagen gel systems to yield a cell density of approximately 3,750 cells/mL.[1]

Preparation of 3D Collagen Gels

3D collagen (type I, rat tail; Dow Corning, Tewksbury, MA) hydrogels were prepared to a final concentration of 2.2 mg/mL according to manufacturer's standard protocol. 10× phosphate buffer saline (PBS; Life Technologies) was mixed with 1 M NaOH (Sigma-Aldrich, St. Louis, MO) (Table 1). Collagen I stock solution with a concentration of 3.3 – 3.7 mg/mL in 0.02 M acetic acid was added to 10× PBS/1 M NaOH solution, thoroughly vortexed, and held in ice. The pH of the solution was tested with a pH spear (Oakton Instruments, Vernon Hills, IL) and incrementally adjusted to 9.2 – 9.5 by addition of 1 - 10 µL 1 M NaOH. Cell culture media containing the desired cell density was slowly added to the collagen to yield a final gel concentration of 2.2 mg/mL and pH of 8.3 – 8.7, and gently mixed prior to polymerization. Custom-made Delrin molds were used to cast

Collagen Gel Concentration	PBS	NaOH	Collagen I, Stock	Cell Solution
2.2 mg/mL	10%	1.4%	56.2%	32.4%

Table 1. Collagen hydrogel composition. v/v% of each component to produce a collagen gel concentration of 2.2 mg/mL.

cylindrical gels of 1.25 mm height and 15 mm diameter. The center base of the mold was sterilized with 99% isopropyl alcohol, coated with a thin layer of vacuum grease, and covered with a 15 mm cover slip. Collagen-cell solution was inserted into the base cavity, sandwiched with a chemically activated, hydrophilic 25 mm top cover slip, and polymerized in a humidified cell incubator at 37°C and 5% CO_2 for 45 minutes. Polymerized gels were removed with a custom-made tool designed to avoid gel damage during removal, and transferred to six-well plates filled with 3 mL of culture media. To maximize nutrient diffusion, the 15 mm cover slip was carefully removed before incubation. Collagen gel cultures were maintained in a humidified cell

incubator at 37°C and 5% CO_2, and culture media was exchanged at 24 h and every 2 days thereafter. Experiments were initiated at day 7 *in vitro* (DIV).

Fluorescence Staining

For all compression experiments, neuron viability and geometry were assessed using calcein AM (Life Technologies), a membrane-permeant dye that is converted from non-fluorescent calcein AM to green-fluorescent calcein upon acetoxymethyl ester hydrolysis by intracellular esterases. Calcein AM has been shown to be well-retained within live cells, and has a high quantum yield.[2,3] Acute alterations in plasma membrane permeability following compressive loading were assessed by evaluating uptake of Alexa Fluor 568 hydrazide (AFH) (Life Technologies), a relatively low molecular weight (730 Da) and cell-impermeant fluorophore used for intracellular labeling experiments.[4] Used extracellularly, AFH cannot infiltrate the cell membrane until pores, approximately the size of AFH, develop due to mechanical insult. Previous studies have shown that this mechanoporation occurs even for significantly larger fluorescent dyes (MW 150 kDa) after mechanical stretch.[5]

Prior to mechanical loading, cell cultures were placed into a Chamlide CMB chamber (Live Cell Instruments Inc., Seoul, South Korea), and incubated with calcein AM (2 μM) and AFH (10 μM diluted with dH_2O) in prewarmed HA + 2% B-27 for 45 minutes. After rinsing with 1× PBS, a 15 mm cover slip was gently placed on top of the gel, and a solution of AFH (10 μM) in HA/B-27 was left in the chamber during mechanical insult and imaging. Studies have shown maintenance of neuronal cell viability and function at ambient CO_2 for up to 48 hours[6–8] in HA/B-27.

Confocal Microscopy and Live Cell Imaging

For cell compression experiments, three-dimensional image volumes of neurons before and immediately after mechanical insult were acquired using a Nikon A-1 confocal system mounted on a Ti-Eclipse inverted optical microscope controlled by Nikon NIS–Elements software (Nikon, Tokyo, Japan). To ensure physiological conditions, temperature within the microscope chamber was controlled at 37°C using a closed-loop heater (Air-Therm heater, World Precision Instruments Inc., Sarasota, FL), and the high-speed compression device was allowed to thermally equilibrate for 2 – 3 hours prior to imaging. A CFI Plan APO VC 60× water immersion objective (NA = 1.2; Nikon) was used for all mechanical compression experiments. Calcein AM-stained neurons were excited with an Argon ion laser (488 nm), and extracellular AFH was excited with a HeNe diode laser (561 nm). Confocal image stacks of 1024 × 1024 × 100 voxels (177 × 177 × 100 μm^3) were recorded with a z-step of 1 μm. Volumes were located at a depth of 100 – 200 μm from the bottom of the collagen gel. For each experiment 6 – 12 cells were imaged directly after mechanical insult, at four hours, and every hour thereafter until somatic lysis or leaking of calcein AM dye coupled with severe morphological changes occurred. Morphological changes and cell death were never observed before four hours in all experiments and thus imaging was begun then to avoid overexposure to the excitation laser.

Reflectance microscopy was used to visualize the fiber structure of the collagen gels. No stain is required and the samples were imaged with an APO 40× water immersion objective (NA = 1.15; Nikon) and Z-step of 0.63 μm, to yield an image volume of 1024 × 1024 × 176 voxels (212 × 212 × 110 μm^3).

Control experiments

Control experiments were conducted to confirm neuron population viability in the collagen hydrogels through 9 DIV (Fig. S1). Culture preparation and maintenance remained the same as described earlier. Again, calcein AM was used as a live cell stain. Ethidium homodimer–1(EthD-1; Life Technologies), a cell-impermeant nucleic acid stain was used to stain dead cells. Upon compromise of the cell membrane, EthD–1 binds to nuclear DNA and, upon laser excitation, fluoresces red. Calcein AM-stained neurons were excited with an Argon ion laser (488 nm), and EthD–1 was excited with a HeNe diode laser (561 nm). Confocal image stacks of 512 × 512 × 200 voxels (635 × 635 × 248 μm^3) were recorded with a z-step of 1.24 μm. Neuron population viability was determined by manual counting aided by flNeuronTool.[9]

Control experiments to assess the cell damage associated with the cell stains, and confocal microscopy imaging were performed under the same conditions as the impact experiments without applying compressive strain (Fig. S1).

3D Cell Compression Device

The 3D cell compression device applies mechanical deformation to neurons cultured in a 3D collagen gel. The primary goals of the cell compression device are (1) uniaxial compression across the 3D collagen cell culture with high strain rates and magnitudes that are characteristic of inertial TBI;[10–13] (2) displacement (as opposed to force) controlled deformation with well-characterized boundary conditions; (3) user defined temporal characteristics of the strain field; and (4) access to cells via confocal microscopy before, during, and after mechanical insult so that the immediate and long term biochemical events are measurable.

The cell compression device consists of a linear voice coil actuator (VCA) (LAS 13-18; BEI Kimco, San Marcos, CA) that is mounted on a custom frame attached to the confocal microscope stage (Fig. 1a). Uniaxial compressive strain is generated by linear motion of the VCA piston in contact with the top cover slip of the collagen gel (Fig. 1b). To ensure normal contact

with the cover slip during deformation, the piston is fitted with a spherical tip. To overcome the limited range of motion of the piston and to increase sample accessibility, the VCA is mounted on a manual z-stage with a full range of motion of 100 mm. The sample chamber is situated in an independent x-y translation stage that is used to precisely center the sample with the piston. Prior to mechanical deformation, a custom cylindrical tool (with same diameter as the sample chamber) with a centered hemispherical cutout (with the same radius as piston tip) is inserted into the x-y stage. The VCA is lowered and the x-y stage is adjusted so that the piston tip fits into the centered cutout. Motion of the piston is produced via a closed loop control system consisting of a servo controller (SilverSterling S2-IG; QuickSilver Controls, Inc., Covina, California) and integrated hall-based displacement transducer. A computer is used to program and communicate with the controller for the desired piston motion. In the study, the VCA was programmed to deliver strain magnitudes of 0.30 and peak strain rates of 10 s^{-1} and 75 s^{-1} (Fig. 1e). Due to the inertia of the system, the actual measured strain differed slightly from the programmed.

Strain Field Characterization

Experimental Parameters

The uniaxial compressive strain field was validated under quasistatic loading on the 3D collagen neuron cultures ($n = 7$). Fluorescent microspheres (0.5 µm in diameter, carboxylate-modified, Life Technologies) were thoroughly mixed at 6 v/v% into the collagen gel during cell culture and maintained in the same conditions as the mechanical impact experiments. At day 7 *in vitro*, collagen cell cultures stained with calcein AM were mounted on the cell compression device and compressed by a 2% strain increment with a 2 minute loading time every 6 minutes to a total strain of 0.38. During the hold time, confocal image volumes of size $512 \times 512 \times 350$ voxels ($212 \times 212 \times 210$ μm^3) were acquired within a depth of 100 – 200 µm from the coverslip using an APO 40× water immersion objective (NA = 1.15, Nikon).

Full-field displacement computation

Full-field displacements of the collagen-embedded microspheres were tracked between loading steps using Fast Iterative Digital Volume Correlation (FIDVC).[14] FIDVC utilizes the iterative deformation method (IDM), which spatially refines the correlation window, allowing for accurate measurement of large, non-linear material displacements, which could arise near the neuron-collagen interface. The final interrogation window spacing, which sets the displacement spatial grid resolution was 8 voxels or 3.3 µm for measurements in $x_1 - x_2$ and 4.8 µm for measurements in x_3. The displacement gradient tensor $\nabla \boldsymbol{u}$, computed with an optimal-9 tap finite difference kernel, was used to calculated the Lagrangian strain field $\mathbf{E}$, given as

$$\mathbf{E} = \frac{1}{2}\left(\nabla \boldsymbol{u} + \nabla \boldsymbol{u}^{\mathrm{T}} + \nabla \boldsymbol{u} \cdot (\nabla \boldsymbol{u})^{\mathrm{T}}\right). \quad (1)$$

The transverse to axial strain ratio ν was computed as the negative ratio of mean transverse strain E_{11} and E_{22} to axial strain E_{33} averaged over all increments N, *i.e.*

$$\nu = -\frac{1}{N}\sum_{n}^{N}\left(\frac{E_{11} + E_{22}}{2E_{33}}\right), \quad (2)$$

and was found to be $\nu = (5.6 \pm 0.8) \times 10^{-2}$.

Spherical Inclusion Problem

To determine the local strain values experienced by cells, a relation between far-field applied strain and cell-experienced strain must be understood. Cells compressed in a 3D matrix can be analytically compared (to first order) to an Eshelby inclusion solution given by Goodier,[15] which describes a spherical inclusion with radius a, Poisson's ratio ν_{I}, and Young's modulus E_{I} in an infinite, isotropic, linear-elastic solid matrix with Poisson's ratio ν_{M} and Young's modulus E_{M} subject to a uniaxial stress at infinity σ^{∞}. The elastic mismatch between the matrix and inclusion gives rise to nonlinear displacements around the interface. The closed-form solution of the radial displacements u_r and tangential displacements u_θ solely due to the elastic mismatch is given by

$$\begin{aligned} u_r &= -\frac{A}{r^2} - \frac{3B}{r^4} + \left(\frac{5-4\nu_{\mathrm{M}}}{1-2\nu_{\mathrm{M}}}\frac{C}{r^2} - \frac{9B}{r^4}\right)\cos 2\theta \\ u_\theta &= -\left(\frac{2C}{r^2} + \frac{6B}{r^4}\right)\sin 2\theta \end{aligned} \quad (3)$$

where A, B, and C are constants determined by equating the displacements and tractions at the interface, *i.e.* $r = a$. The values of the constants are

$$
\begin{aligned}
A =& -a^3 \frac{\sigma^\infty}{8\mu_\mathrm{M}} \frac{\mu_\mathrm{M} - \mu_\mathrm{I}}{(7-5\nu_\mathrm{M})\mu_\mathrm{M} + (8-10\nu_\mathrm{M})\mu_\mathrm{I}} \\
& \times \frac{(1-2\nu_\mathrm{I})(6-5\nu_\mathrm{M})2\mu_\mathrm{M} + (3+19\nu_\mathrm{I} - 20\nu_\mathrm{M}\nu_\mathrm{I})\mu_\mathrm{I}}{(1-2\nu_\mathrm{I})2\mu_\mathrm{M} + (1+\nu_\mathrm{I})\mu_\mathrm{I}} \\
& + \frac{\sigma^\infty}{4\mu_\mathrm{M}} \frac{\left((1-\nu_\mathrm{M})\frac{1+\nu_\mathrm{I}}{1+\nu_\mathrm{M}} - \nu_\mathrm{I}\right)\mu_\mathrm{M}}{(1-2\nu_\mathrm{I})2\mu_\mathrm{M} + (1+\nu_\mathrm{I})\mu_\mathrm{I}} \\
B =& a^5 \frac{\sigma^\infty}{8\mu_\mathrm{M}} \frac{\mu_\mathrm{M} - \mu_\mathrm{I}}{(7-5\nu_\mathrm{M})\mu_\mathrm{M} + (8-10\nu_\mathrm{M})\mu_\mathrm{I}} \\
C =& a^3 \frac{\sigma^\infty}{8\mu_\mathrm{M}} \frac{5(1-2\nu_\mathrm{M})(\mu_M - \mu_\mathrm{I})}{(7-5\nu_\mathrm{M})\mu_\mathrm{M} + (8-10\nu_\mathrm{M})\mu_\mathrm{I}}
\end{aligned}
\tag{4}
$$

where the matrix shear modulus μ_M and inclusion shear modulus μ_I are related to the Young's modulus and Poisson's ratio by

$$
\begin{aligned}
\mu_\mathrm{M} &= \frac{E_\mathrm{M}}{2(1+\nu_\mathrm{M})} \\
\mu_\mathrm{I} &= \frac{E_\mathrm{I}}{2(1+\nu_\mathrm{I})}.
\end{aligned}
\tag{5}
$$

The full-field displacements in the matrix are obtained by superposing the displacements in equation (3) with the solution for uniaxial compression/tension along x_3,

$$
\begin{aligned}
u_r &= \frac{\sigma^\infty a}{2E_\mathrm{M}}\left((1-\nu_\mathrm{M}) + (1+\nu_\mathrm{M})\cos 2\theta\right) \\
u_\theta &= -\frac{\sigma^\infty a}{2E_\mathrm{M}}(1+\nu_\mathrm{M})\sin 2\theta.
\end{aligned}
\tag{6}
$$

To compare between the experimentally determined displacement field around the cell and the Eshelby solution, an inclusion ($a = 5$ voxels, $\nu_\mathrm{I} = 0.49$) in a matrix ($\nu_\mathrm{M} = 0.06$) is subjected to a remote stress given by

$$
\sigma^\infty = \frac{E_\mathrm{M}}{1+\nu_\mathrm{M}}\left(E_{33}^\infty + \frac{\nu_\mathrm{M}}{1-2\nu_\mathrm{M}}(E_{11}^\infty + E_{22}^\infty + E_{33}^\infty)\right). \tag{7}
$$

E_{33}^∞ is the far-field strain applied by the cell compression device, and E_{11}^∞ and E_{22}^∞ are the strain components that arise due to the Poisson effect. The displacement along the loading axis u_3 is computed after transforming equations (3) – (6) to Cartesian coordinates for varying ratios of $E_\mathrm{I}/E_\mathrm{M}$. It is important to note that there are values of $E_\mathrm{I}/E_\mathrm{M}$ that produce well-known deformations; $E_\mathrm{I}/E_\mathrm{M} = 0$ is the Eshelby solution of a spherical cavity subject to a remote stress; $E_\mathrm{I}/E_\mathrm{M} = 1$ is the solution for uniform strain; and $E_\mathrm{I}/E_\mathrm{M} = \infty$ is the Eshelby solution for a rigid inclusion subject to a remote stress. Qualitative comparison of the experimental local displacement field around neurons to the Eshelby inclusion solution for different ratios of $E_\mathrm{I}/E_\mathrm{M}$ showed good agreement within a range of 0.5 to 2.0 (Fig. S2), suggesting that the imposed far-field strain is not significantly affected by a local elastic mismatch, which is consistent with research on other cell types.[16] Thus, cells are assumed to locally undergo the imposed far-field strain during impact.

Image Preprocessing

Converting raw confocal images of neurons to quantitative metrics that can be used for injury analysis requires a multiphase image processing routine (Fig. S3). The 3D confocal micrographs were split to isolate the calcein AM and Alexa Fluor Hydrazide channels independently and were converted to .tif files, which were then loaded into MATLAB (Natick, MA) for preprocessing. Images were rigidly translated to correct for experimental drift by maximizing the cross-correlation between an image and the image at the next time point using the calcein AM channel.

A median filter with a $3 \times 3 \times 3$ voxel window size was then applied twice for noise reduction, followed by a gamma correction with a coefficient of 0.7 ($I(\boldsymbol{x}) = I(\boldsymbol{x})^{0.7}$) for contrast enhancement. To remove background noise, all intensity values below the mode of the image were set to zero.

Building Neuron Tree Structure

The tree data structure, commonly used in graph theory, is a powerful way to describe the morphology of a neuron. In this study, the neuron tree structure, $T = \{V, E\}$, is composed of a set of n nodes or vertices V connected by a set of $n-1$ edges E that are directed away from a root that has no parent node (Fig. S4a). Nodes are classified by the degree, d, or number of children that directly follow, *i.e.* $d = 1$, extension; $d > 1$, fork; and $d = 0$, end or leaf. For quantitative geometric and injury analysis, nodes and edges can store important information (*e.g.* position, radius, and strain). Implementation of the neuron tree allows for easy and efficient traversal along paths by eliminating repetition.

To build a tree structure, each calcein AM image was exported as .tiff format and loaded into Neuron Studio, a software that performs semi-automatic tracing and reconstruction of neuron structures from confocal images.[17] From a manually selected seed point at the soma center, Neuron Studio automatically thresholds and skeletonizes the image with a topology and geometry-preserving algorithm that computes the medial axis.[18,19] Branch diameters are estimated at each node along the skeleton or medial axis using the Rayburst sampling algorithm.[18,19] In this algorithm, the image is trilinearly interpolated along ray vectors that are cast from a node, and are allowed to grow until a specified intensity is reached. This intensity value is automatically adjusted by Neuron Studio according to the local intensity distribution of the image. Final node diameters are determined via rayburst spheres, estimated by computing the median ray vector distances for each node, which are less affected by local surface irregularities.

The Neuron Studio tracing and reconstruction procedure produced a tree structure of the neuron (Fig. S4b), which was carefully edited for validity, *i.e.* extensions automatically built from other neurons in the image were carefully removed using the built-in 3D viewer. To prevent undersampling, the "Discretization Ratio" parameter, which sets the sampling density of nodes, was set to the minimum value of zero, producing the most dense nodal connectivity. Additionally, spurious extensions that were produced from image noise were removed by setting the "Minimum Length" parameter to 5 μm. The image channel of the cell-impermeant Alexa Fluor Hydrazide dye was manually traced since the neuronal extensions were not as well defined. In most cases, the tree consisted of a soma and one to two short neurites.

After editing, the neuron trees were exported as 'swc' format, read into MATLAB, and converted to a tree structure for quantitative injury analysis. Functions that were not custom written for a specific application came from the TREES toolbox,[20] a graph theory library specifically for neuron tree structures in MATLAB.

Defining local coordinate frames for neuron structures

Defining quantitative injury metrics requires the definition of a local coordinate frame. Each neurite extension, if treated like a smooth parameterized space curve, $\boldsymbol{x}(s)$, can be described by an orthonormal Frenet frame, $\boldsymbol{f}(s)$.[21] At each point on the curve, the Frenet frame is defined as

$$\boldsymbol{f}(s) = \{\boldsymbol{t}(s), \boldsymbol{n}(s), \boldsymbol{b}(s)\}, \tag{8}$$

where $\boldsymbol{t}(s)$, $\boldsymbol{n}(s)$, and $\boldsymbol{b}(s)$ are the tangent, normal, and binormal unit vectors (Fig. S5a). They are mathematically expressed as

$$\begin{aligned} \boldsymbol{t}(s) &= \frac{\boldsymbol{x}'(s)}{\|\boldsymbol{x}'(s)\|}, \\ \boldsymbol{n}(s) &= \frac{\boldsymbol{t}'(s)}{\|\boldsymbol{t}'(s)\|} = \frac{\boldsymbol{x}'(s) \times (\boldsymbol{x}''(s) \times \boldsymbol{x}'(s))}{\|\boldsymbol{x}'(s)\| \|\boldsymbol{x}''(s) \times \boldsymbol{x}'(s)\|}, \\ \boldsymbol{b}(s) &= \boldsymbol{t}(s) \times \boldsymbol{n}(s) = \frac{\boldsymbol{x}'(s) \times \boldsymbol{x}''(s)}{\|\boldsymbol{x}'(s) \times \boldsymbol{x}''(s)\|}. \end{aligned} \tag{9}$$

The curvature, $\kappa(s)$, and twist, $\tau(s)$, of $\boldsymbol{x}(s)$ are related to $\boldsymbol{t}(s)$, $\boldsymbol{n}(s)$, and $\boldsymbol{b}(s)$, and their derivatives by the Frenet-Serret system of differential equations, but are not utilized in this study as they are dependent on the curve fit third derivative $\boldsymbol{x}'''(s)$, which is not guaranteed to be smooth for our chosen cubic spline fitting. Although the Frenet frame $\boldsymbol{f}(s)$ can be computed along the extensions of the cell, its rotation about $\boldsymbol{t}(s)$ can behave erratically at degenerate points where the curvature vanishes, *e.g.* inflection points or straight segments. To circumvent this issue for injury analysis, the injury metrics proposed in this study will be invariant to rotation about $\boldsymbol{t}(s)$.

Practical Implementation of a discrete local coordinate frame for neuron structures

In practice, using equations (8) to define a local coordinate frame is numerically challenging for many reasons. Extensions are not made of smooth curves, but piecewise linear functions that can sharply change direction at certain nodes due to image noise. Additionally, node sampling is highly dependent on the tracing parameters in Neuron Studio. To circumvent these issues, a series of processing steps was performed before computing the local coordinate frame.

Tree resampling: To begin the processing procedure, the neuron tree was resampled using the 'resample_tree' function in the TREES toolbox, which redistributes the nodes at a user defined equal distance away from each other. The resampling distance was chosen as the 25th percentile of all edge lengths, such that most geometric features are preserved.
Tree smoothing: After resampling, trees were smoothed to eliminate sharp corners using Taubin smoothing, an algorithm commonly used to remove noise on polygonal meshes while preserving salient shape features.[22–24] To adapt this algorithm for trees, first the discrete Laplacian of $\boldsymbol{x}$ is computed for each extension node (d = 1) with the expression

$$\Delta \boldsymbol{x}_i(s) = \frac{1}{2}\left(\boldsymbol{x}_{i-1}(s) - \boldsymbol{x}_i(s)\right) + \frac{1}{2}\left(\boldsymbol{x}_{i+1}(s) - \boldsymbol{x}_i(s)\right). \tag{10}$$

Geometrically, $\Delta \boldsymbol{x}_i$ are the average positions of neighboring nodes to the ith node. In general there are more advanced weighting schemes for the discrete Laplacian operator. However, since the tree is resampled at equidistant points, the effects would be minimal. A Laplacian filter is then applied by updating the position $\boldsymbol{x}$ with $\Delta \boldsymbol{x}$, given by the equation

$$\boldsymbol{x}(s) = \boldsymbol{x}(s) + \lambda \Delta \boldsymbol{x}(s) \tag{11}$$

where λ is a scale factor that controls the extent of smoothing, and is $0 < \lambda < 1$. The Laplacian filter must be applied iteratively many times to produce significant smoothing. However, doing so would result in large tree contraction or shrinking. To overcome this issue, Taubin incorporated an expansion step following each contraction step. Mathematically, the expansion step is similar to equation (11), but λ is replaced with a negative scale factor μ such that $\mu < -\lambda$. The resulting transfer function of the expansion and shrinking operation has a pass-band frequency given by

$$k_{PB} = \lambda^{-1} + \mu^{-1} > 0. \tag{12}$$

All trees in this study were smoothed 100 times with $\lambda = 0.63$ and $k_{PB} = 0.1$, chosen from.[24]
Computing discrete tangent: Having sufficiently smooth trees allows for computation of $\boldsymbol{f}(s)$, for which there are many numerical methods.[25,26] In this study, a piecewise cubic spline (C^2 differentiable) was fit to a set of tree paths using the "spline" function in MATLAB. Each path was defined as set of extension type nodes that connect a root and fork, root and end, fork and fork, or fork and end. As opposed to other methods, cubic splines return a set of third order polynomial coefficients that can be analytically evaluated to compute the geometric features at any point.

Image Post-Processing

To accurately tag the formation of blebs or to determine when cell death occurred, all image information outside of the tree was removed (Fig. S6a). To perform this masking procedure, connected nodes in the tree were isolated and converted to a nodal mask by setting interior voxels to 0 and exterior voxels to 1 (Fig. S6 b and c). For each nodal mask, a convex hull (CH) was computed and superimposed with the previous nodal mask using an OR logical operation to construct the CH of the whole cell (Fig. S6d). The final image mask was computed by dilating the CH of the whole cell using a Euclidean distance transform, the 'bwdist' function, where all values within a distance of 32 voxels were set to 0 and outside were set to 1 (Fig. S6e).

Quantitative Injury Analysis

Local Strain Transformation

The strain that neurons experience is highly dependent on the local orientations of their neurites. For example, a neurite that is aligned with the principal loading axis will experience more axial strain than one that is transversely aligned with the loading axis. Analysis for this orientation-dependent deformation draws from classic laminate theory and strain rosette calculations, where the global strain or stress is transformed to coordinates of the material fibers or strain gauges. For both of these cases, the analysis is simplified using plane stress assumptions, which is not valid for neurons. To extend the theory into 3D, the peak far-field strain $\mathbf{E}^{\infty}$ must be rotated to the direction of each edge in the neuron tree. The transformation is expressed as

$$\mathbf{E}^{(\boldsymbol{f})} = \mathbf{Q} \cdot \mathbf{E}^{\infty} \cdot \mathbf{Q}^{\mathrm{T}}. \tag{13}$$

where $\mathbf{E}^{(\boldsymbol{f})}$ is strain matrix in the local basis, $\mathbf{Q}$ is the rotation matrix. $\mathbf{E}^{\infty}$ is given from the strain applied by the cell compression device and the transverse to axial strain ratio ν as

$$\mathbf{E}^{\infty} = 0.3 \cdot \begin{bmatrix} \nu & 0 & 0 \\ 0 & \nu & 0 \\ 0 & 0 & -1 \end{bmatrix}. \tag{14}$$

Using the Frenet frame ($\boldsymbol{f}$) along $\boldsymbol{x}(s)$, $\mathbf{Q}$ is given by

$$\mathbf{Q} = \begin{bmatrix} \boldsymbol{b}\cdot\boldsymbol{e}_1 & \boldsymbol{b}\cdot\boldsymbol{e}_2 & \boldsymbol{b}\cdot\boldsymbol{e}_3 \\ \boldsymbol{n}\cdot\boldsymbol{e}_1 & \boldsymbol{n}\cdot\boldsymbol{e}_2 & \boldsymbol{n}\cdot\boldsymbol{e}_3 \\ \boldsymbol{t}\cdot\boldsymbol{e}_1 & \boldsymbol{t}\cdot\boldsymbol{e}_2 & \boldsymbol{t}\cdot\boldsymbol{e}_3 \end{bmatrix} \tag{15}$$

where $\boldsymbol{e}$ is the global Cartesian coordinate frame, $\boldsymbol{t}$ is the local tangent vector, and $\boldsymbol{e}_3$ is the loading axis. Applying the rotation in equation (15) to $\mathbf{E}^{(f)}$, equation (13) becomes

$$\mathbf{E}^{(f)} = \begin{bmatrix} \boldsymbol{b}\cdot\mathbf{E}^{\infty}\cdot\boldsymbol{b} & \boldsymbol{b}\cdot\mathbf{E}^{\infty}\cdot\boldsymbol{n} & \boldsymbol{b}\cdot\mathbf{E}^{\infty}\cdot\boldsymbol{t} \\ & \boldsymbol{n}\cdot\mathbf{E}^{\infty}\cdot\boldsymbol{n} & \boldsymbol{n}\cdot\mathbf{E}^{\infty}\cdot\boldsymbol{t} \\ & & \boldsymbol{t}\cdot\mathbf{E}^{\infty}\cdot\boldsymbol{t}. \end{bmatrix} \tag{16}$$

Since $\boldsymbol{t}$ is the most uniquely defined geometric quantity in the system, we compute the axial strain, defined as

$$E_c = \boldsymbol{t}\cdot\mathbf{E}^{\infty}\cdot\boldsymbol{t}, \tag{17}$$

(Fig. S5 d and e). Because the orthonormal vectors $\boldsymbol{n}$ and $\boldsymbol{b}$ are non-unique in our system, we define an average axial shear strain as

$$E_s = \sqrt{(\boldsymbol{n}\cdot\mathbf{E}^{\infty}\cdot\boldsymbol{t})^2 + (\boldsymbol{b}\cdot\mathbf{E}^{\infty}\cdot\boldsymbol{t})^2}. \tag{18}$$

After equations (17) and (18) are computed for every point along a neuron, the mean axial strain is defined as

$$\langle E_c \rangle = \frac{1}{L}\int_{\boldsymbol{x}(s)} E_c(s)\,ds \tag{19}$$

and the mean axial shear strain is defined as

$$\langle E_s \rangle = \frac{1}{L}\int_{\boldsymbol{x}(s)} E_s(s)\,ds \tag{20}$$

where L is the total length of the neuron, *i.e.* $L = \int_{\boldsymbol{x}(s)} ds$.

Injury Assessment

Bleb Formation: For each neuron that exhibited clear blebbing, a time series of maximum intensity projection images was created. The image at the time point at which severe blebbing occurred was loaded into flNeuronTool, a software for 3D visualization and proofreading of neuronal morphologies.[9] All blebs were tagged using the ‘probing’ mode and imported into MATLAB for analysis. The maximum compressive axial strain and axial shear strain of all nodes within a specified radial distance of each bleb were computed. This distance was equal to $3.35 \pm 0.73\mu$m, computed as the mean bleb radius of 26 blebs as manually recorded from three representative neurons.

Membrane Poration: AFH trees were loaded into MATLAB and each node sphere along the neuron tree was converted into a binary mask. Neurons were considered porated if the mean intensity within the mask increased to the mean background intensity. The same analysis was performed for the membrane poration controls.

Cell Death Assessment: Time of cell death was assessed as the timepoint at which somatic lysis or a combination of leaking of live cell dye calcein AM and severe morphological deformities occurred. Leaking of calcein AM fluorescence has been shown to correlate with other cell death markers.[27]

Bivariate Compressive Axial Strain Fitting and Probability Density Estimation: The scatter data of the mean compressive axial strain $\langle E_c \rangle$ as a function of time at cell death t_d was fit to a bivariate normal distribution using the minimum covariance determinant (MCD) estimator[28,29] implemented in the LIBRA MATLAB library.[30] MCD is commonly used in bivariate fitting since outliers in the data can greatly influence the fitting. The major and minor axis of the 95 % ellipse were computed from the eigenvalues of the covariance matrix of the fit. To qualitatively confirm the validity of the bivariate normal distribution, the 2D probability density was computed using the commonly used non-parametric adaptive algorithm for kernel density estimation.[31]

References

1. Berthiaume, F. & Morgan, J. R. *Methods in Bioengineering: 3D Tissue Engineering* (Artech House, 2014).
2. De Clerck, L. S., Bridts, C. H., Mertens, A. M., Moens, M. M. & Stevens, W. J. Use of fluorescent dyes in the determination of adherence of human leucocytes to endothelial cells and the effect of fluorochromes on cellular function. *J. Immunol. Methods* **172**, 115–124 (1994).

3. Papadopoulos, N. G. *et al.* An improved fluorescence assay for the determination of lymphocyte-mediated cytotoxicity using flow cytometry. *J. Immunol. Methods* **177**, 101–111 (1994).
4. Spence, M. T. & Johnson, I. D. *The molecular probes handbook: a guide to fluorescent probes and labeling technologies* (Life Technologies Corporation, 2010).
5. Geddes, D. M., Cargill, R. S. & LaPlaca, M. C. Mechanical stretch to neurons results in a strain rate and magnitude-dependent increase in plasma membrane permeability. *J. Neurotrauma* **20**, 1039–1049 (2003).
6. Brewer, G. J. & Price, P. J. Viable cultured neurons in ambient carbon dioxide and hibernation storage for a month. *Neuroreport* **7**, 1509–1512 (1996).
7. Kivell, B. M., McDonald, F. J. & Miller, J. H. Method for serum-free culture of late fetal and early postnatal rat brainstem neurons. *Brain Res. Protoc.* **6**, 91–99 (2001).
8. Brewer, G. J. & LeRoux, P. D. Human primary brain tumor cell growth inhibition in serum-free medium optimized for neuron survival. *Brain Res.* **1157**, 156–166 (2007).
9. Ming, X. *et al.* Rapid reconstruction of 3d neuronal morphology from light microscopy images with augmented rayburst sampling. *PLoS One* **8**, e84557 (2013).
10. Margulies, S. S. & Thibault, L. E. An analytical model of traumatic diffuse brain injury. *J. Biomech. Eng.* **111**, 241–249 (1989).
11. Margulies, S. S., Thibault, L. E. & Gennarelli, T. A. Physical model simulations of brain injury in the primate. *J. Biomech.* **23**, 823–836 (1990).
12. Margulies, S. S. & Thibault, L. E. A proposed tolerance criterion for diffuse axonal injury in man. *J. Biomech.* **25**, 917–923 (1992).
13. Meaney, D. F. *et al.* Biomechanical analysis of experimental diffuse axonal injury. *J. Neurotrauma* **12**, 689–694 (1995).
14. Bar-Kochba, E., Toyjanova, J., Andrews, E., Kim, K.-S. & Franck, C. A fast iterative digital volume correlation algorithm for large deformations. *Exp. Mech.* **55**, 261–274 (2015).
15. Goodier, J. N. Concentration of stress around spherical and cylindrical inclusions and flaws. *J. appl. Mech* **55**, 39–44 (1933).
16. Solon, J., Levental, I., Sengupta, K., Georges, P. C. & Janmey, P. A. Fibroblast adaptation and stiffness matching to soft elastic substrates. *Biophys. J.* **93**, 4453–4461 (2007).
17. Rodriguez, A. *et al.* Automated reconstruction of three-dimensional neuronal morphology from laser scanning microscopy images. *Methods* **30**, 94–105 (2003).
18. Wearne, S. *et al.* New techniques for imaging, digitization and analysis of three-dimensional neural morphology on multiple scales. *Neuroscience* **136**, 661–680 (2005).
19. Rodriguez, A., Ehlenberger, D. B., Hof, P. R. & Wearne, S. L. Rayburst sampling, an algorithm for automated three-dimensional shape analysis from laser scanning microscopy images. *Nature protocols* **1**, 2152–2161 (2006).
20. Cuntz, H., Forstner, F., Borst, A. & Häusser, M. One rule to grow them all: a general theory of neuronal branching and its practical application. *PLoS Comput. Biol.* **6**, e1000877 (2010).
21. Kreyszig, E. *Differential Geometry* (Dover Publications, 1991).
22. Taubin, G. A signal processing approach to fair surface design. In *Proceedings of the 22nd annual conference on Computer graphics and interactive techniques*, 351–358. ACM (Association for Computing Machinery (ACM), 1995).
23. Taubin, G. Curve and surface smoothing without shrinkage. In *Computer Vision, 1995. Proceedings., Fifth International Conference on*, 852–857. IEEE (Institute of Electrical & Electronics Engineers (IEEE), 1995).
24. Taubin, G. *et al.* Geometric signal processing on polygonal meshes. *Eurographics State of the Art Reports* **4** (2000).
25. Hu, S., Lundgren, M. & Niemi, A. J. Discrete frenet frame, inflection point solitons, and curve visualization with applications to folded proteins. *Physical Review E* **83**, 061908 (2011).
26. Struik, D. J. *Lectures on classical differential geometry* (Courier Corporation, 2012).
27. Bratosin, D., Mitrofan, L., Palii, C., Estaquier, J. & Montreuil, J. Novel fluorescence assay using calcein-am for the determination of human erythrocyte viability and aging. *Cytometry Part A* **66**, 78–84 (2005).
28. Rousseeuw, P. J. Least median of squares regression. *J. Am. Stat. Assoc.* **79**, 871–880 (1984).

29. Rousseeuw, P. J. & Driessen, K. V. A fast algorithm for the minimum covariance determinant estimator. *Technometrics* **41**, 212–223 (1999).

30. Verboven, S. & Hubert, M. Libra: a matlab library for robust analysis. *Chemometr. Intell. Lab.* **75**, 127–136 (2005).

31. Botev, Z., Grotowski, J., Kroese, D. *et al.* Kernel density estimation via diffusion. *Ann. Stat.* **38**, 2916–2957 (2010).

Figures

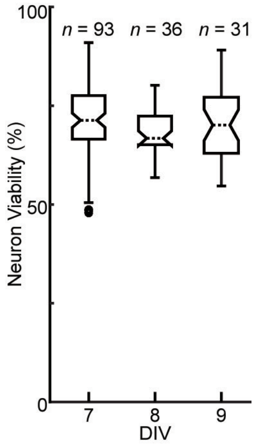

Figure 1. Control viability data. Box and whisker plot of the control viability for 3D neuron culture populations at 7 DIV ($n = 93$ volumes), 8 DIV ($n = 36$), and 9 DIV ($n = 31$). Dotted bands: median; top and bottom boxes: upper and lower quartiles; whiskers: 5th and 95th percentiles. No significant difference between data sets using two-way ANOVA.

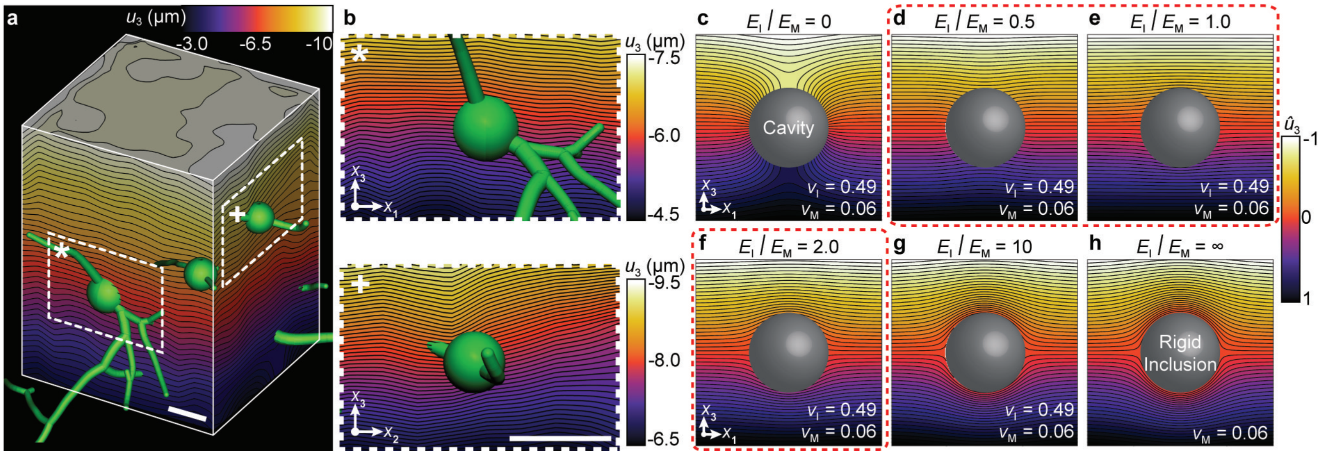

Figure 2. Displacement field around a neuron with inclusion solution comparison (**a**) Contour plot of the vertical displacement, u_3, in the collagen matrix surrounding the neurons (green). Scale bar, 50 µm. (**b**) Zoomed-in view of the data subset (white box) shown in a. Scale bar 20 µm. (**c**–**h**) Analytical solution of the matrix displacement around a spherical inclusion (gray) with radius $a = 5$ voxels, Young's Modulus E_I, and Poisson's Ratio $\nu_I = 0.49$ in an infinite matrix with Young's Modulus E_M and Poisson's Ratio $\nu_M = 5.6 \times 10^{-2}$ subject to a uniaxial far-field stress. The ratio E_I/E_M was varied from 0 to ∞, with d-f in qualitative agreement with a-b.

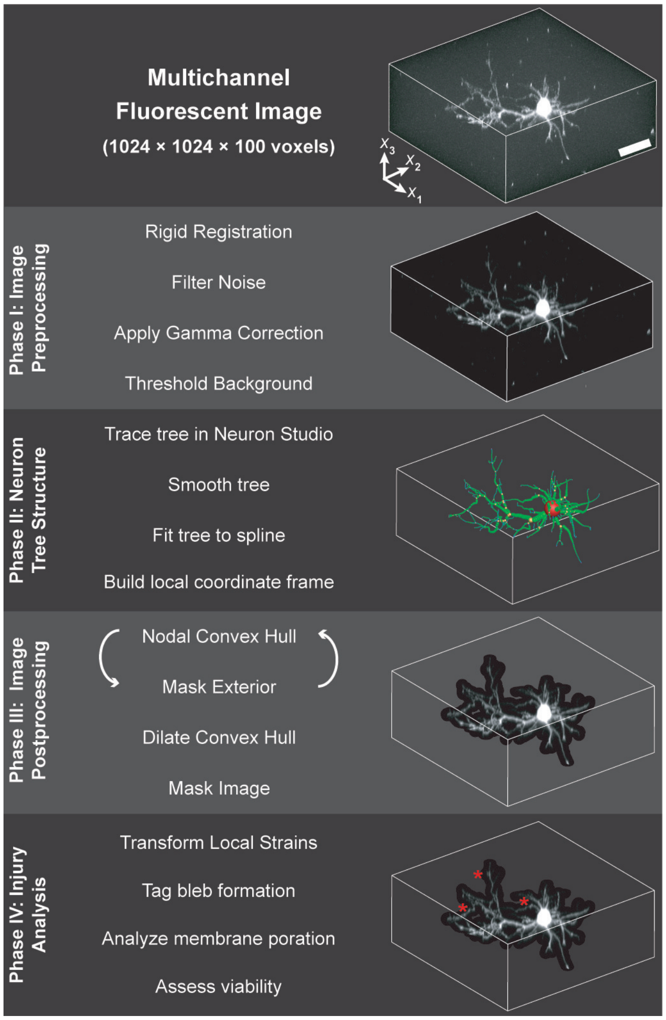

Figure 3. Main image processing steps for neuronal strain injury analysis. Scale bar, 40 µm.

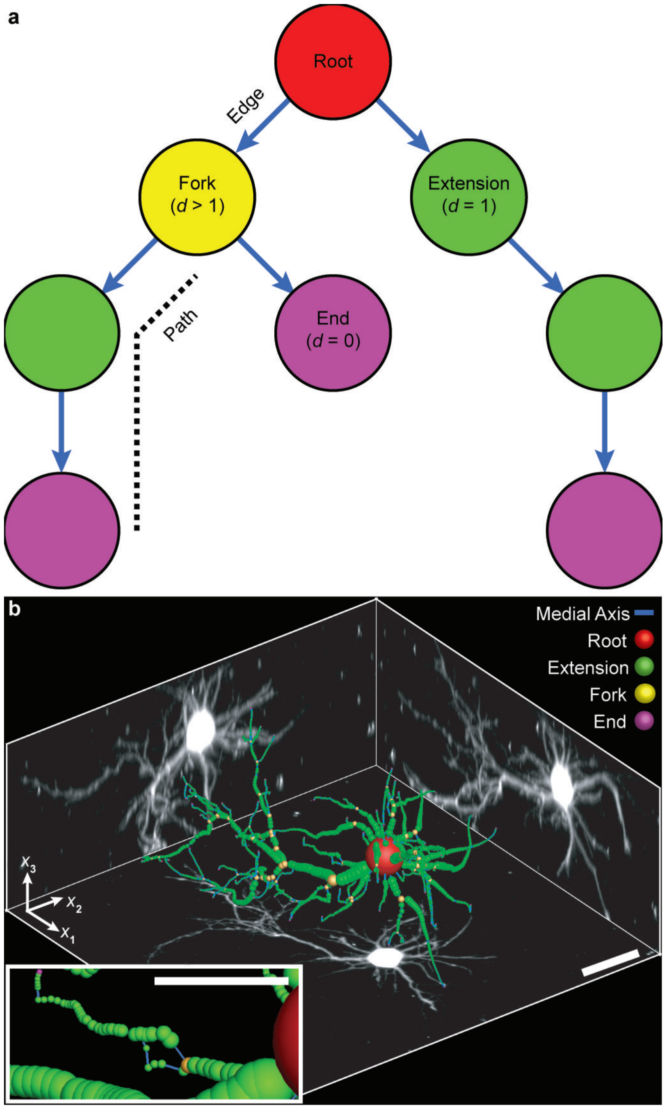

Figure 4. Building a tree structure from a volumetric image of a neuron. (**a**) Tree data structure with a set of nodes connected by edges directed away from a root (red). Nodes are categorized by the number of directly connected children or degree (d); $d = 1$ (green), extension; $d > 1$, fork (yellow); and $d = 0$, end (magenta). Tree branches are followed along paths and defined by a sequence of connected nodes without repetition. (**b**) Neuron tree projected in 3D. Scale bar, 20 µm. Inset scale bar, 5 µm.

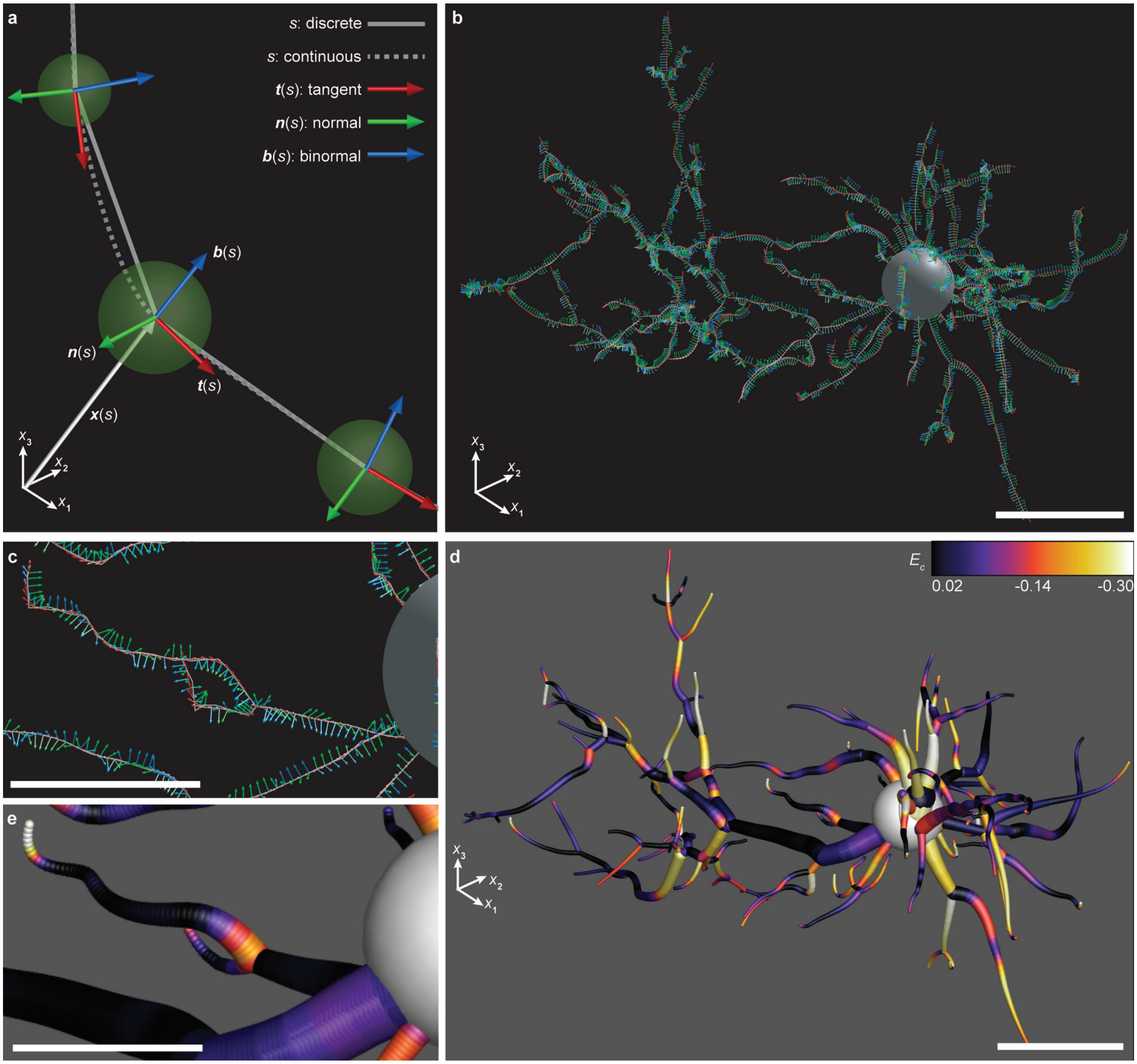

Figure 5. Local coordinate system and strain transformation on a neuron. (**a**) Schematic representation of local Frenet frame $\boldsymbol{f}(s) = \{\boldsymbol{t}(s), \boldsymbol{n}(s), \boldsymbol{b}(s)\}$ on nodes (green) at positions $\boldsymbol{x}(s)$ along smoothed paths (dotted line). (**b**) Frenet frame $\boldsymbol{f}(s)$ computed for a neuron tree. Scale bar 20 μm. (**c**) Zoomed-in view of B. Scale bar 10 μm. (**d**) Axial strain computed along a neuron tree using equation (17). Scale bar, 20 μm. (**e**) Zoomed-in view of D. Scale bar 10 μm.

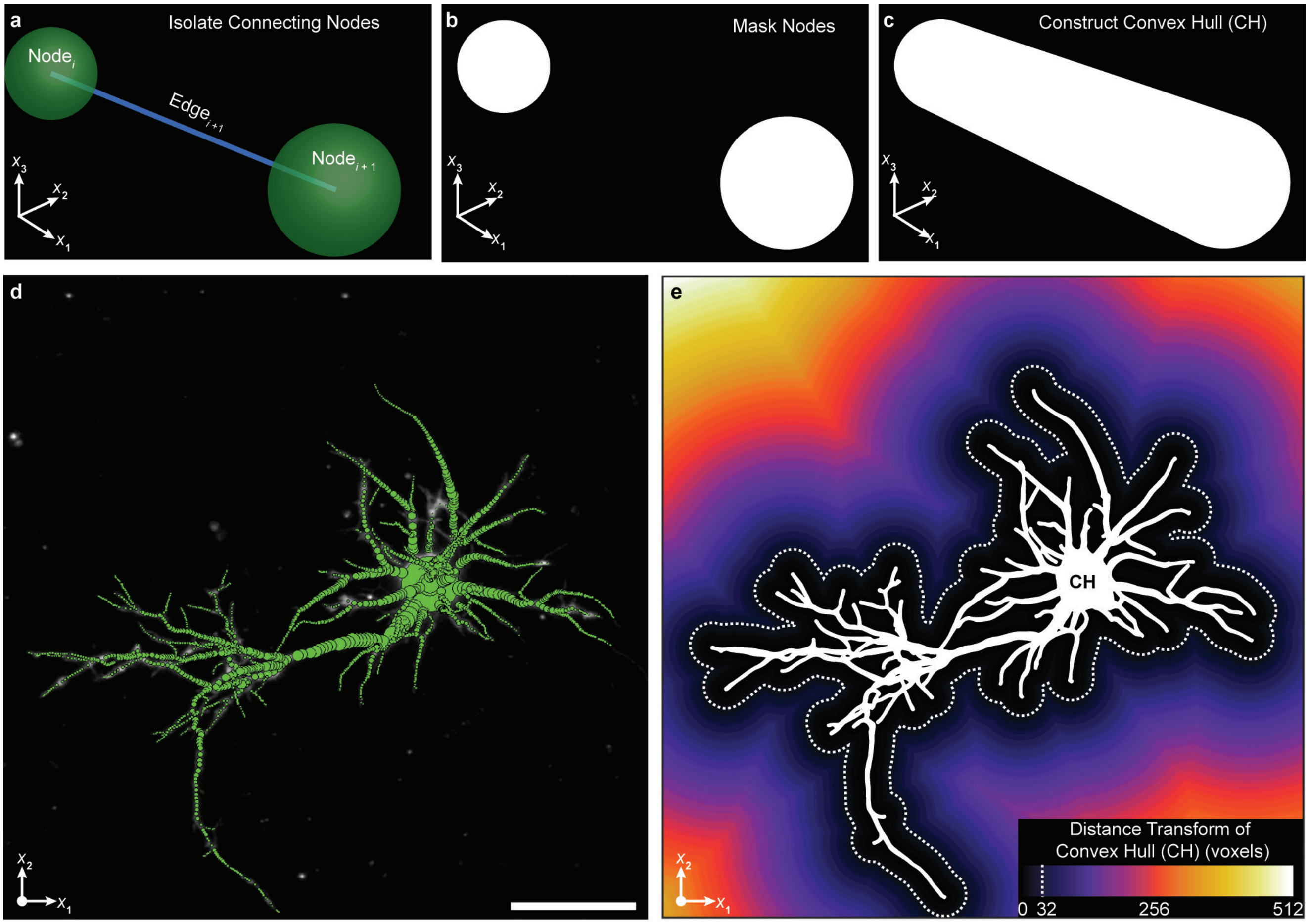

Figure 6. Image post-processing to remove information outside the neuron. (**a**) Neighboring nodes connected by an edge. (**b**) Binary mask generated from nodes. (**c**) The convex hull (CH) generated between nodes is computed and superimposed on the entire tree. (**d**) Neuron tree projected in 3D before CH is applied. Scale bar, 40 μm. (**e**) CH applied to the entire neuron. The final image mask was computed by setting all voxels within 32 voxels (dotted) away from CH to 1.
